# Novel predator-induced phenotypic plasticity by hemoglobin and physiological changes in the brain of *Xenopus tropicalis*

**DOI:** 10.3389/fphys.2023.1178869

**Published:** 2023-06-06

**Authors:** Tsukasa Mori, Kazumasa Machida, Yuki Kudou, Masaya Kimishima, Kaito Sassa, Naoko Goto-Inoue, Ryuhei Minei, Atsushi Ogura, Yui Kobayashi, Kentaro Kamiya, Daiki Nakaya, Naoyuki Yamamoto, Akihiko Kashiwagi, Keiko Kashiwagi

**Affiliations:** ^1^ Nihon University College of Bioresource Sciences, Fujisawa, Japan; ^2^ Department of Computer Bioscience, Nagahama Institute of Bio-Science and Technology, Nagahama, Japan; ^3^ Milk. Inc, Tokyo, Japan; ^4^ Department of Animal Sciences, Graduate School of Bioagricultural Sciences, Nagoya University, Nagoya, Japan; ^5^ Hiroshima University Amphibian Research Center, Hiroshima, Japan

**Keywords:** phenotypic plasticity, *Xenopus tadpoles*, novel predators, threat response, hemoglobin

## Abstract

Organisms adapt to changes in their environment to survive. The emergence of predators is an example of environmental change, and organisms try to change their external phenotypic systems and physiological mechanisms to adapt to such changes. In general, prey exhibit different phenotypes to predators owing to historically long-term prey-predator interactions. However, when presented with a novel predator, the extent and rate of phenotypic plasticity in prey are largely unknown. Therefore, exploring the physiological adaptive response of organisms to novel predators is a crucial topic in physiology and evolutionary biology. Counterintuitively, *Xenopus tropicalis* tadpoles do not exhibit distinct external phenotypes when exposed to new predation threats. Accordingly, we examined the brains of *X. tropicalis* tadpoles to understand their response to novel predation pressure in the absence of apparent external morphological adaptations. Principal component analysis of fifteen external morphological parameters showed that each external morphological site varied nonlinearly with predator exposure time. However, the overall percentage change in principal components during the predation threat (24 h) was shown to significantly (*p* < 0.05) alter tadpole morphology compared with that during control or 5-day out treatment (5 days of exposure to predation followed by 5 days of no exposure). However, the adaptive strategy of the altered sites was unknown because the changes were not specific to a particular site but were rather nonlinear in various sites. Therefore, RNA-seq, metabolomic, Ingenuity Pathway Analysis, and Kyoto Encyclopedia of Genes and Genomes analyses were performed on the entire brain to investigate physiological changes in the brain, finding that glycolysis-driven ATP production was enhanced and *ß*-oxidation and the tricarboxylic acid cycle were downregulated in response to predation stress. Superoxide dismutase was upregulated after 6 h of exposure to new predation pressure, and radical production was reduced. Hemoglobin was also increased in the brain, forming oxyhemoglobin, which is known to scavenge hydroxyl radicals in the midbrain and hindbrain. These suggest that *X. tropicalis* tadpoles do not develop external morphological adaptations that are positively correlated with predation pressure, such as tail elongation, in response to novel predators; however, they improve their brain functionality when exposed to a novel predator.

## 1 Introduction

Phenotypic plasticity is the ability of the same genotype to exhibit different phenotypes induced by physiological responses to environmental change (Pigliucci, 2001; Price et al., 2003; West-Eberhard, 2003). Therefore, it is an excellent marker of adaptation (Spitze, 1992; Schoeppner and Relyea, 2005; Kishida et al., 2009; Nunes et al., 2013). Morphological and physiological changes induced by predator–prey interactions represent a type of phenotypic plasticity (Brönmark and Miner, 1992; Tollrian, 1995; Tollrian and Harvell, 1999; Weisser et al., 1999; Gilbert, 2009; Jarrett, 2009), offering evidence of predation stress in prey. Therefore, investigating phenotypic plasticity in terms of physiological and external morphological changes in response to a novel predator could help elucidate the initial steps of consequent evolutionary processes that are important to organisms. For example, anuran tadpoles, belonging to the lower trophic levels of the food web, live in predator-abundant [e.g., dragonfly larvae (McCollum and Leimberger, 1997; Van Buskirk et al., 1997; Kishida et al., 2010)] water bodies. As a morphological adaptation, the tail depth of some species has increased to facilitate faster swimming to escape predators, thereby considerably enhancing their survival (Johnson et al., 2015). Bulgy formations in *Rana pirica* tadpoles are developed in response to native predators, such as the larval *Hynobius retardatus* (Hokkaido salamander), to hinder consumption (Kishida and Nishimura, 2004). Phenotypic plasticity caused by exposure to native predators, as seen in the predator-prey interactions of *R. pirica* and *H. retardatus*, has revealed noticeable adaptations by prey species during co-evolution (Van Buskirk and Relyea, 1998; Kishida and Nishimura, 2005). Similarly, novel predators present a threat to prey, and such encounters could initiate novel physiological changes in living organisms and further evolutionary adaptations.

In a previous study, we exposed *X. laevis* tadpoles to predatory larvae of the non-native Tōhoku salamander (*Hynobius lichenatus*) (Mori et al., 2017). Although *Xenopus laevis* tadpoles did not exhibit behavioral inhibition or morphological response, such as the bulgy formations observed in *R. pirica*, they exhibited other morphological responses, such as significant growth of the tail muscle and increased tail length, which considerably increased their average swimming speed (Mori et al., 2017).

Predation stress is also known to elevate corticosterone concentrations in tadpoles, activating the neuroendocrine stress axis (Middlemis Maher et al., 2013). Corticosterone induces the hypothalamic-pituitary-adrenal axis to adapt to environmental changes induced by acute stress (Kinlein et al., 2019). Therefore, a brain-centered fear response has been predicted, and previous studies reported changes to the brain transcriptome when threatened by predators (Jöngren et al., 2010; Sanogo et al., 2011). However, adaptations of the brain cannot be comprehensively understood based on the expression patterns of a few genes.

Therefore, we performed RNA sequencing (RNA-seq) of the whole brain of *X. laevis* tadpoles exposed to novel predation stress in a previous study and investigated changes in signal transduction pathways using ingenuity pathway analysis (IPA).

The results showed that *X. laevis* tadpoles did not exhibit noticeable phenotypic changes, such as the bulgy formation observed in *R. pirica* tadpoles; however, certain signaling pathways in the brain were suppressed following a 6 h exposure to predation threat, with many pathways being re-activated after 24 h and then re-suppressed after 10 days. In the experiment, we found that the axonal extension from the nostrils to the diencephalon noticeably increased after 24 h of predation exposure, and there were changes in neural networks in the brain in response to novel predation within 24 h (Mori et al., 2020). Further, in the experiment, we found that hemoglobin gene expression was increased in the *X. laevis* tadpoles exposed to predation stress (unpublished data).

*X. tropicalis* is generally known to have a much smaller habitat than *X. laevis*, as its range is restricted to the equatorial area. In addition, salamanders reportedly do not exist on the African continent (Allen and Schlager, 2005) or are only found in the northern part of the continent, suggesting that the habitat of *X. tropicalis* is different from that of salamanders. Therefore, exposing *X. tropicalis*. (native to Africa) to an unfamiliar predator (e.g., Japanese salamander, *H. retardatus*). is a suitable system for studying phenotypic plasticity in predator–prey interactions using novel predators.

In this study, *X. tropicalis* tadpoles were exposed to *H. retardatus* larvae and changes in external morphology at 15 sites, including the head and tail, were analyzed to determine whether the tadpoles showed tail elongation in response to predation stress, as in the case of *X. laevis*. Furthermore, to determine whether the increased expression of hemoglobin genes observed in *X. laevis* tadpoles also occurs in the brain of *X. tropicalis* in response to a novel predator, we performed RNA sequencing (RNA-seq) using whole brain of *X. tropicalis*, and then identified differentially expressed genes (DEGs), which were subjected to Kyoto Encyclopedia of Genes and Genomes (KEGG: https://www.kegg.jp/kegg/kegg1.html) pathway enrichment analysis (Kanehisa and Goto, 2000; Kanehisa et al., 2016). We further investigated changes in signal transduction pathways using ingenuity pathway analysis (IPA), analyzed brain metabolites by capillary electrophoresis–mass spectrometry (CE-MS), and attempted to determine the significance of physiological adaptation strategies, for instance, whether exposure to a novel predator also increases hemoglobin in the brains of *X. tropicalis* tadpoles.

## 2 Materials and methods

### 2.1 Ethical statement

The authors declare that the research presented in this manuscript complied with the Animal Welfare Guidelines for Journal Publication. All animal housing and experimental protocols were in compliance with the Japanese Government Animal Protection and Management Law (No. 105) and Japanese Government Notification on Feeding and Safekeeping of Animals (No. 6). All experimental protocols were approved by the Japanese University Regulations for the Conduct of Animal Experiments, and the experiments were conducted in accordance with Japanese University protocols (https:// www.nihon-u.ac.jp/uploads/files/20221116152822.pdf). In Japan, no special ethics document is required for experiments on amphibians (https://www.mext.go.jp/b_menu/hakusho/nc/06060904.htm), and Nihon University approved this and our experiments with amphibians. The American Veterinary Medical Association (AVMA) Guidelines for Euthanasia of Animals (2020) and the guidelines of Science Council of Japan (https://www.scj.go.jp/ja/info/kohyo/pdf/kohyo-20-k16-2.pdf) were used for veterinary best practices on the anesthesia and euthanasia of animals. Since live *Xenopus* was used in this study, we conducted the experiments in adherence to ARRIVE guidelines (https://arriveguidelines.org).

### 2.2 Experimental animals and design

The *X. tropicalis* tadpoles used in this study were obtained from an inbred line prepared by the Amphibian Research Center of Hiroshima University, Japan. Twenty tadpoles of similar size (stage 51) were randomly selected from the holding tank and were placed in 2 L glass aquaria (25 × 10 × 8 cm) containing 1 L tap water treated with activated charcoal (Tsurumi Coal, Kanagawa, Japan) for 24 h. Tadpole developmental stages were determined based on the *X. laevis* stage series (https://www.xenbase.org/entry/anatomy/alldev.do). Tadpoles were fed Sera Micron (Sera, Heinsberg, Germany) and were housed at 25°C throughout the experiment. Detailed information on the experimental animals and design is provided in the Supplementary Methods.

**FIGURE 1:**
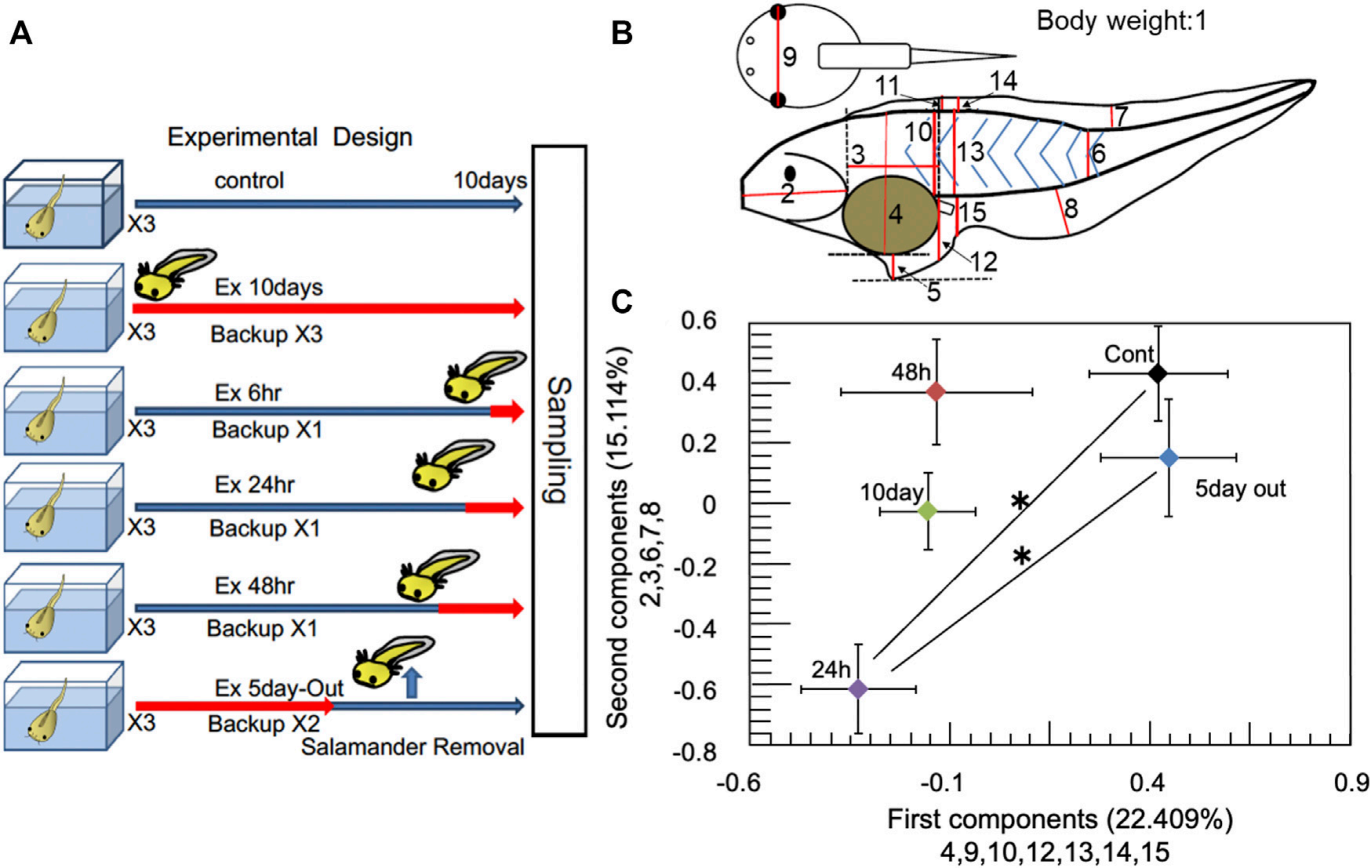
Experimental design and multiscale analysis. **(A)** Experimental design. Control represents no predator exposure. Ex 10 days, Ex 6, Ex 24, and Ex 48 h represent that one salamander was reared with 20 *Xenopus tropicalis* tadpoles in an aquarium for 10 days, 6, 24, and 48 h before sampling, respectively. Ex 5 days-Out represents that the salamander was removed after 5 days, and that the tadpoles were reared for the next 5 days without predation threat. Red arrows represent the period that the salamander was present. Each treatment group had three replicate aquaria. **(B)** Body parameters of the tadpoles measured for principal component analysis. Body weight was measured as parameter 1. Data were analyzed using ImageJ (ver1.53e: https://imagej.nih.gov/ij/index.html). **(C)** Results of the averaged PCA. Ex 10 days, Ex 48, Ex 24, Ex 5 days-Out, and control are shown as 10 days, 48, 24 h, 5 days out, and C, respectively. *X*-axis shows the main measurement factors chosen for the first principal component (numbers 4, 9, 10, 12, 13, 14, and 15; see Figure 2B). *y*-axis shows the main measurement sites selected for the second component (2, 3, 6, 7, and 8). The bars on the *x*-axis and *y*-axis for each sample indicate the standard error. multivariate analysis of variance (MANOVA) was performed on the principal component analysis (PCA) results. * denotes statistically significant (*p* < 0.05) differences for both the first and second components. Error bars indicate standard errors. See Supplementary Methods and Supplementary Table S4 for detailed information.

Tadpoles were selected for experimentation based on a previously described randomized protocol (Mori et al., 2017) (see Figure 1A; Supplementary Methods). Prior to the experiment, approximately 200–300 tadpoles were randomly selected from approximately 1,500–1,800 tadpoles and placed in a 20 L tank (45 × 15 × 30 cm). From these randomized tadpoles, 20 (at stage 51) were randomly selected from the 20 L tank containing 200‒300 tadpoles and placed in a experimental 2 L glass aquaria. The selection of tadpoles from the 20 L tank for the various experimental groups was performed randomly. Six predator exposure conditions were used, including an unexposed control, and 10-day (Ex 10 days), 48 h (Ex 48 h), 24 h (Ex 24 h), and 6 h (Ex 6 h) exposures. Another modified group was also used, with a predator for 5 days and none for the next 5 days (Ex 5 days-Out). Eighteen aquaria were set up for predation-exposed and control tadpole groups. Three, one, one, one, and two extra aquaria were prepared as backups for the Ex 10 days, Ex 48, Ex 24, Ex 6 h, and Ex 5 days-Out groups, respectively. Thus, each treatment group included three replicates and a variable number of backups. Twenty additional tadpoles of similar size (stage 51) were placed in a 2 L glass aquaria as backups. All aquaria (including backups) were randomly arranged and separated using cardboard as blinders. Tadpoles killed by predation in each experimental tank were removed and tadpoles were added from backup tanks to maintain experimental density. The aquaria were placed under natural day–night conditions in an experimental room at 25°C. Salamander (*H. retardatus*) egg masses were sampled from natural ponds in Asahikawa and Nayoro, Hokkaido, Japan (see Supplementary Figure S1 in the Supplementary Methods). The egg masses were then reared to the larval stage. Predation experiments were initiated by introducing one salamander larva to each aquarium containing 20 tadpoles. To minimize the loss of tadpoles during the predation experiments, we replaced salamander larvae daily with backups kept in a holding tank. From the three tanks of each experimental group, *X. tropicalis* specimens were transferred to a single large container (10 L), where they were mixed. *X. tropicalis* specimens were then randomly sampled from the mixed samples, placed in a transparent plastic case for measurement, and photographed with a camera (Casio, Exilim). The images were used for measurement of morphological changes (Supplementary Table S1) by principal component analysis (PCA).

*X. tropicalis* specimens were placed on paper towels and removed water, and then they were weighed. Data of photographs were analyzed using ImageJ (ver1.53e: https://imagej.nih.gov/ij/index.html).

### 2.3 RNA extraction

Total RNA was extracted from the brain for RNA-seq. To this end, three tadpoles from each replicate aquarium were pooled as a single sample (See experimental design Figure 1A). Thus, nine tadpoles were used per treatment group; the tadpoles were soaked in 1% ice cold Ethyl 3-aminobenzoate methanesulfonate (Sigma-Aldrich) for anesthesia. Brain tissue was dissected under a Leica (Wetzlar, Germany) stereomicroscope, soaked in RNAlater (RNA stabilization reagent, Qiagen, Hilden, Germany), stored overnight at 4°C, and maintained at −80°C until RNA extraction. Total RNA was purified from each sample using a RNeasy Mini Kit (Qiagen), following the manufacturer’s protocol. Total RNA quality was confirmed at an absorbance ratio A_260_/A_280_ (range, 1.7–2.1). 18S to 28S ribosomal RNAs were visually observed using gel electrophoresis.

### 2.4 Library preparation and sequencing

Library preparation and sequencing of extracted total RNA were performed by the Beijing Genomics Institute (BGI) Group (Shenzhen, China). Details are provided in the Supplementary Methods.

### 2.5 Transcriptome analysis and IPA

Differential expression analysis was performed using the R-based package EdgeR (v.3.8.6) (https://bioconductor.org/packages/release/bioc/html/edgeR.html) (Robinson et al., 2010). EdgeR filters out low-expression genes, while retaining those with >1 count per million (CPM) in at least two samples. After normalizing the trimmed mean of the M-values, DEGs were extracted from 170 pairwise comparisons of the control vs. the Ex 6, Ex 24, Ex 48 h, Ex 10 days, and Ex 5 days-out experimental groups using an exact test with FDR <0.05. These extracted DEGs were visualized on a heat map. IPA (Qiagen) was used to identify the functional networks of genes expressed in the brain. Details are provided in the Supplementary Methods.

### 2.6 Capillary electrophoresis time-of-flight mass spectrometry (CE-TOF-MS) analysis of *X. tropicalis* tadpole brains

Tadpoles were anesthetized in an ice water bath and decapitated. Then, they were immediately dissected on ice. The brains were excised under a microscope, placed in a tube, and instantly frozen with liquid nitrogen. Brains from two tadpoles were combined to form a single sample because the brain of one tadpole is not enough for metabolite analysis. Three samples were treated as one experimental group. Details on the CE-MS procedure were previously published (Soga et al., 2006) and are provided in the Supplementary Methods.

### 2.7 Measurement of free radicals and glucose content in *X. tropicalis* tadpoles

*X. tropicalis* tadpoles were sampled and soaked in ice cold 1% Ethyl 3-aminobenzoate methanesulfonate for anesthesia, followed by removal of excess water using paper towels. Next, they were weighed, immediately frozen in liquid nitrogen, and maintained at −80°C until analysis. Tadpoles were homogenized using a Polytron homogenizer (Kinematica, Switzerland) at maximum speed on ice. The mixture was then centrifuged at ×12,000 g for 15 min at 4°C. Then, 20 μL supernatant was used for free radical analysis using the d-ROMs test (Trotti et al., 2001) (Wismerll, Tokyo, Japan). Another 50 μL supernatant was used to estimate glucose levels using the Dri-Chem system (FDC3030, Fujifilm, Tokyo, Japan).

### 2.8 Real-time PCR

Real-time PCR (Rotor-Gene thermal cycler, Qiagen) was used to determine the gene expression profiles of *sod3*, *psph*, *g6pc3*, *hba3*, *alas2*, *fah*, *psat1*, and *pygm* using RNA extracted from the brain. The primers for these genes are presented in Supplementary Table S2. PCR was run for 35 cycles as follows: incubation at 95°C for 5 min, annealing at 95°C for 5 s, and extension at 60°C for 10 s. Expression levels were calculated based on the ^ΔΔCT^ method. Details are provided in the Supplementary Methods.

### 2.9 Statistical analysis of PCA

Fifteen body parts of *X. tropicalis* (Figure 1B) were compared among the five experimental groups, including controls. Relationships between the measured variables and the five groups were subjected to PCA using SPSS (version 20). The mean first and second principal components of each experimental group were plotted against those of all samples, which included 30 control, 65 Ex10 days, 27 Ex 5 days-Out, 28 Ex 48 h, and 44 Ex 24 h specimens. MANOVA was performed on the PCA results, followed by multiple comparisons of the five components. The first principal component was tested using Dunnett’s T3 test, as the test of homogeneity was rejected. The second principal component was tested using the Bonferroni *post hoc* test. Detailed information on the statistical analysis is provided in the Supplementary Methods.

### 2.10 Visualization of hemoglobin using a hyperspectral camera

Hemoglobin from *X. tropicalis* larvae was measured using a CosmosEye HSC1702 camera (Hokkaido Satellite Co., Ltd., Japan) mounted on a Leica (Wetzlar, Germany) stereomicroscope. Hemoglobin was visualized by analyzing the generated hyperspectral images with the normalized difference vegetation index (Lausch et al., 2013). The ratio of oxygen to hemoglobin in tadpole brains was analyzed, following the methods of Tetschke et al. (2016), to calculate oxygen saturation with normalized absorption spectra. Detailed information is provided in the Supplementary Methods.

### 2.11 Immunohistochemistry for brain

Tadpoles anesthetized with ice cold 1% Ethyl 3-aminobenzoate methanesulfonate were fixed with 4% paraformaldehyde solution overnight at 4°C. The samples were then prepared for immunostaining using sequential dehydration with ethanol and were stored in 100% ethanol at −80°C until use. For immunostaining of the brain, tadpoles were depigmented with hydrogen peroxide solution (methanol:H_2_O_2_, 2:1) and then rehydrated with ethanol and phosphate-buffered saline with Tween 20 (PBSTW) (1X PBS +0.1% Tween 20). The samples were washed with PBSTW(x) (PBSTW+5% dimethyl sulfoxide) and blocked with PBSTW(x) + 10% normal goat serum (NGS). They were then incubated overnight with primary hemoglobin subunit epsilon 1 (HBE1) anti-rabbit antibody (PA5-77997; Thermo Fisher) diluted (1:500) in PBSTW(x) and 1% NGS at 4°C. After washing the primary antibody with PBSTW(x) and 1% NGS three times for 5 min, the samples were incubated with the Alexa Fluor 546 anti-rabbit secondary antibody diluted (1:1,000) in PBSTW(x) and 1% NGS overnight at 4°C. After washing the secondary antibody with PBSTW(x) and 0.1% NGS, brains were removed from the tadpoles and embedded in DPX mountant for histological analysis (Sigma-Aldrich) and were observed using confocal microscopy (Leica TCS SP8).

### 2.12 Statistics and reproducibility

RNA-seq read data were analyzed using AfterQC (v.2.7) (Chen et al., 2017). Data were mapped to the *X. tropicalis* reference genome using HISAT2 (v.2.1.0) (Fifield et al., 2015). To analyze gene alignment, the sequence alignment/map (SAM) file, which is in text format, must first be converted to its binary counterpart, binary alignment/map (BAM), which is a highly tractable format for SAMtools (v.1.9) (Abdelmohsen et al., 2009). Reads mapped to each gene were counted using StringTie (v.1.3.4) (Pertea et al., 2016), and the differential expression analysis was performed in R. IPA (Qiagen) was used to identify functional networks of genes, whereby only those with fold changes >1.5 were analyzed (Content version: 48207413). Statistical analyses of PCR and CE-MS results as well as hemoglobin, glucose concentrations, and ROS levels, were performed using a one-wayANOVA, followed by a Bonferroni *post hoc* test or Dunnett’s T3 test.

## 3 Results

### 3.1 Morphological changes and PCA

Mean values of the first and second components for the control, Ex 24, Ex 48 h, Ex 10 days, and Ex 5 days-Out groups are shown in Supplementary Figure S2 in Supplementary Methods. Factor loadings for PCA and statistical analysis data are provided in Supplementary Tables S3, S4, respectively. PCA using the fifteen body parts showed that the external morphology sites in the first component differed from those in the second component (Figure 1C). Similarly, the external morphology sites from the third to the fifth components also changed, indicating that the external morphology site changed nonlinearly with stress time in response to predation stress.

### 3.2 DEGs and KEGG analysis

**FIGURE 2:**
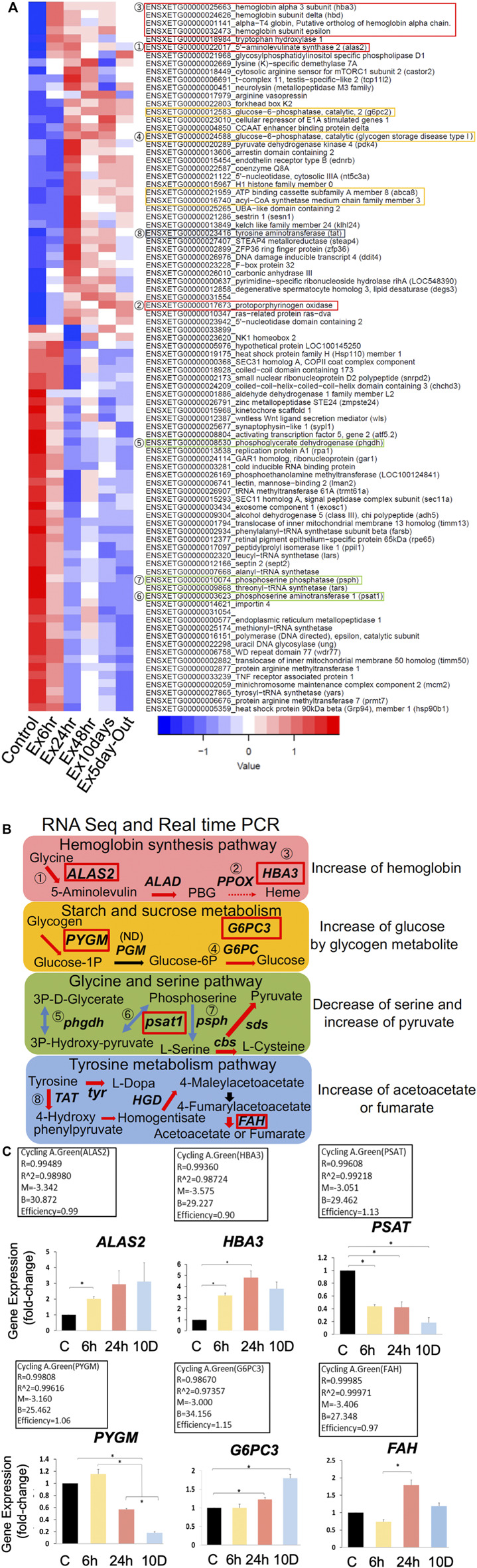
(Continued).

DEGs between the six groups were identified based on a false discovery rate (FDR) < 0.05 (Figure 2A). Major metabolic pathways were predicted using KEGG analysis. DEGs were mined from RNA-seq data (fold change >1.5), and four major pathways were predicted (Figure 2B). Genes with no numbers in Figure 2B were checked in the full dataset (DDBJ BioProject database: PRJDB 14079). Notably, the predicted pathways indicated increases in hemoglobin, glycogenesis, and pyruvate production to reduce serine, and acetoacetate production through tyrosine metabolism. Gene expression levels were quantified using real-time polymerase chain reaction (PCR; Figure 2C). The results were similar to those of RNA-seq, except for glycogen phosphorylase (*pygm*). Overall, PCR efficiency was >0.90, indicating that RNA-seq results were reasonably reliable.

### 3.3 Glycolysis, tricarboxylic acid cycle (TCA), and *ß*-oxidation pathways

**FIGURE 3:**
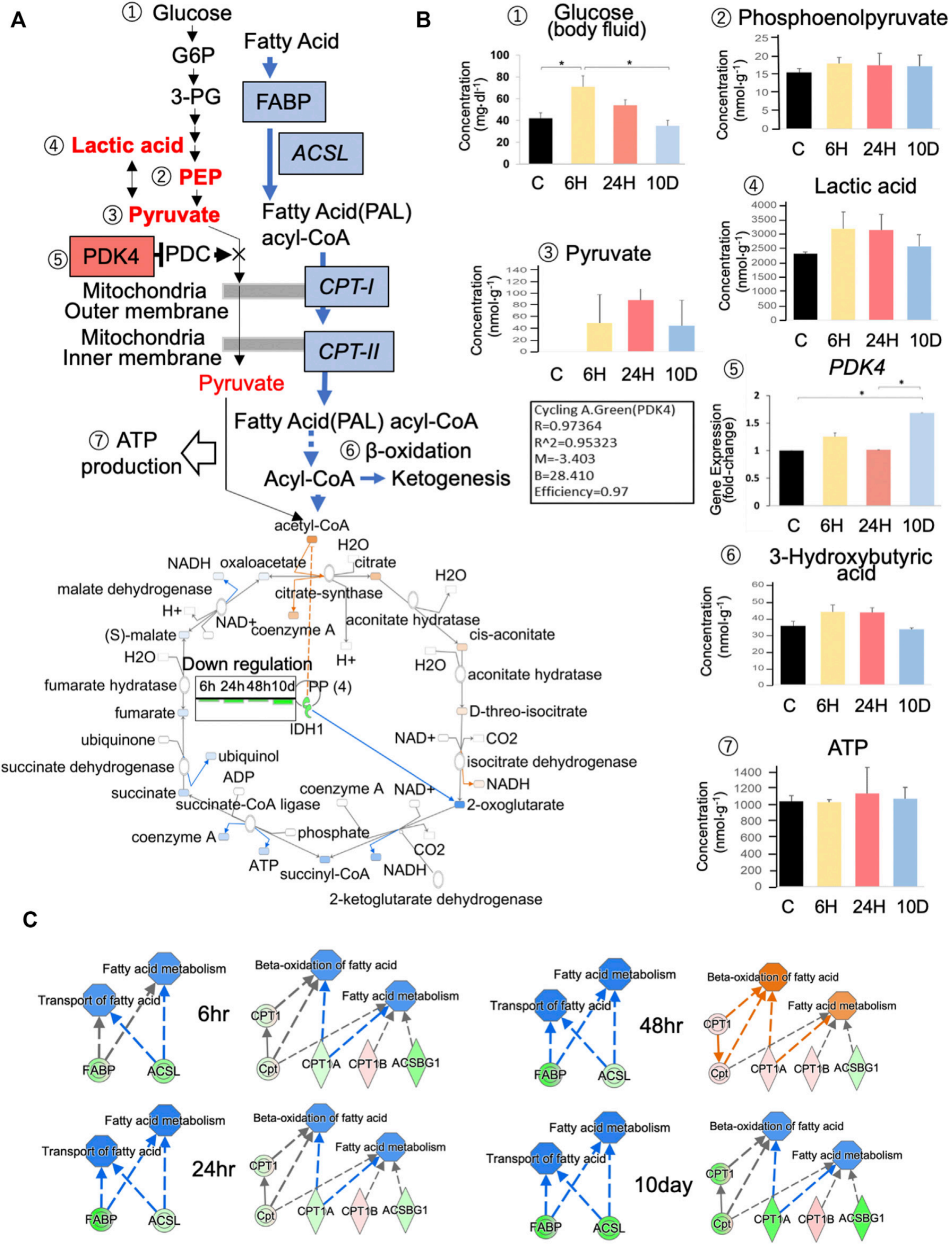
Effects of predation stress on energy production systems. **(A)** Metabolites and enzymes related to glycolysis, the tricarboxylic acid cycle (TCA), and *ß*-oxidation in the brain of *Xenopus tropicalis* tadpoles subjected to predator stress. Ingenuity pathway analysis (IPA) (Content v.48207413) was used to create Figure 4A. Concentration of metabolites and gene expression levels are examined in **(B)**. Numbers ①–⑦ in **(A)** correspond to those shown in **(B)**. Ex 10 days, Ex 24, Ex 6 h, and Control are shown as 10 D, 24, 6 h, and C, respectively. CE–MS and Dri-Chem systems were used to measure glucose content in brain tissue and body fluids, respectively. **(C)** Predictions of changes in the expression of fatty acid-binding proteins (*fabp1–9*), long-chain fatty-acid-CoA synthase (*acsl*), and carnitine *O*-palmitoyltransferase (*cpt*) type I–II using IPA. Blue and red represent down- and upregulation, respectively; green and pink represent down- and upregulation in RNA-seq data, respectively; blue and orange arrows represent down- and upregulation, respectively. * *p* < 0.05 represented statistical significance based on one-way ANOVA, followed by a Bonferroni post hoc test or Dunnett’s T3 test.

We analyzed glycolysis and *ß*-oxidation pathways (Figure 3A) using CE-MS, real-time PCR, and IPA predictions based on RNA-seq data. While CE–MS cannot be used to detect uncharged glucose in the brain, glucose concentrations in body fluid after a 6-h exposure to predation stress were significantly elevated (67.1 mg dl^−1^; Figure 3B). *pdk4* blocks the pyruvate dehydrogenase complex and halts pyruvate metabolization via the TCA cycle; however, its gene expression decreased after a 6-h exposure to predation stress (Figure 2A) but tended to be slightly higher than that of the control based on PCR (Figure 3B). Pyruvate was not detected in the control immediately before *pdk4* downregulation, but was elevated within 6 h and up to 10 days after predation stress (Figure 3B). Lactic acid also increased within 6 h of predation stress, whereas isocitrate dehydrogenase (*idh1*) expression was downregulated from 6 h to 10 days afterward (Figure 3A). Notably, ATP concentrations were similar in all groups (*p* = 0.970; Figure 3B).

The *ß*-oxidation pathway was also investigated using IPA based on RNA-seq data (Figure 3C). Fatty acid-binding proteins bind to fatty acids, which are processed into fatty acid acyl-CoA under the action of the long-chain acyl-CoA synthetase (acsl). Acyl-CoA is then transported by carnitine *O*-palmitoyltransferase (*cpt*) type I–II to the mitochondria for *ß*-oxidation (Figure 3A). Fatty acid-binding proteins, acsl, cpt-1, and cpt-2, were downregulated from 6 h to 10 days after predation stress. However, at 48 h of predation stress, cpt-1, cpt-2, *ß*-oxidation, and fatty acid metabolism were predicted to be upregulated. The levels of 3-hydroxybutyric acid, a product of ketogenesis, generally increased following 6 h of predation stress but decreased to control levels after 10 days; however, these results were not statistically significant (Figure 3B).

### 3.4 CE–MS analysis

**FIGURE 4:**
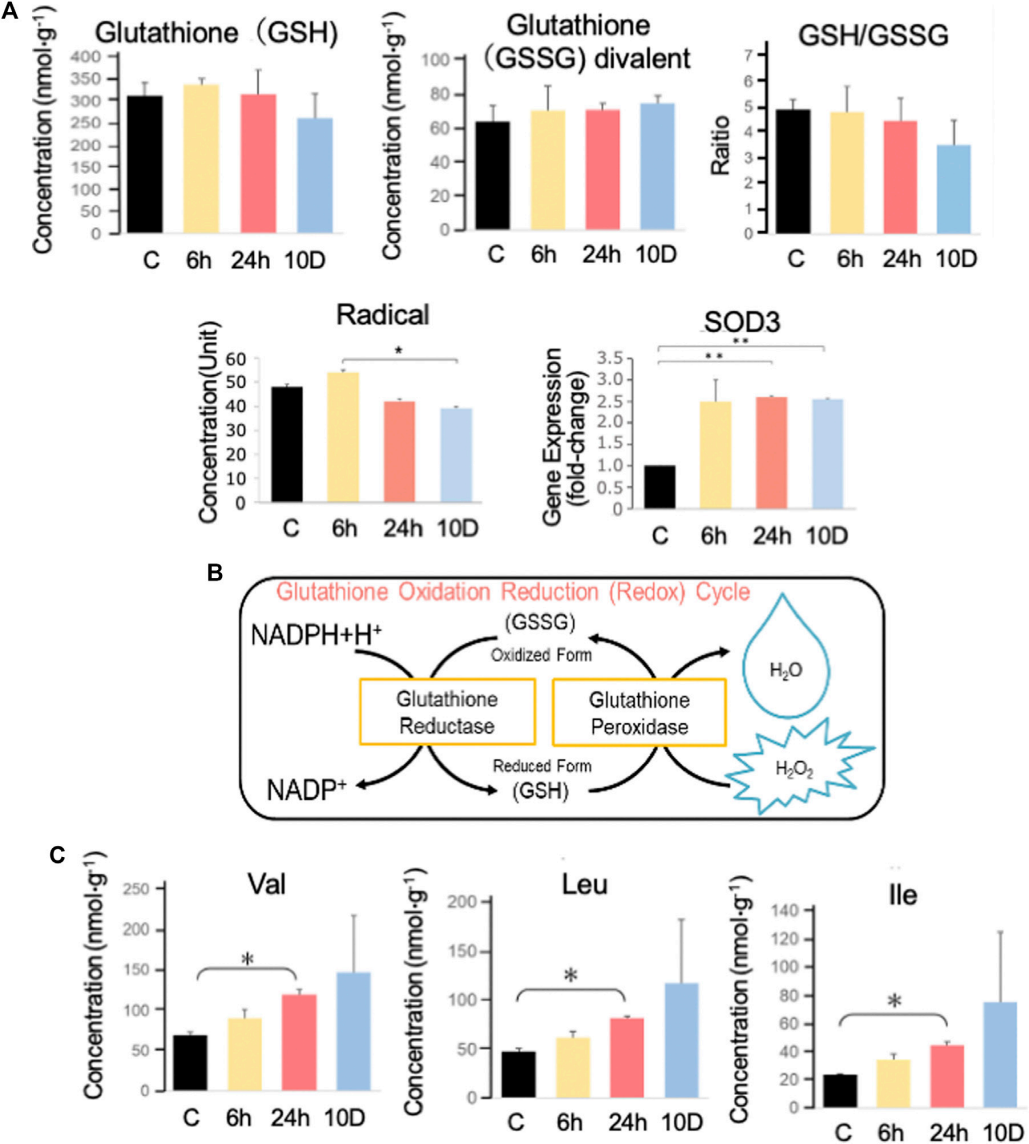
Capillary electrophoresis–mass spectrometry (CE–MS) analysis. **(A)** GSH and GSSG in *Xenopus tropicalis* brains were measured using CE–MS analysis, and the GSH/GSSG ratio was determined. SOD3 expression was determined using real-time PCR, whereas reactive oxygen species (ROS) were measured using the d-ROMs test. **(B)** Illustration of the glutathione oxidation-reduction cycle. **(C)** Val, Leu, and Ile were measured using CE–MS analysis. Ex 10 days, Ex 48, Ex 24, Ex 6 h, and control are shown as 10 D, 48, 24, 6 h, and C, respectively. *p < 0.05 represented statistical significance based on one-way ANOVA, followed by a Bonferroni *post hoc* test or Dunnett’s T3 test.

The physiological condition of tadpoles was also inferred based on metabolome data (Figure 4A). The reduced form of glutathione (GSH), which scavenges radicals, slightly decreased with increasing predation stress (Figure 4B). Conversely, its oxidized form (GSSG) slightly increased with predation stress. GSH contributes slightly to the removal of superoxide, and the GSH/GSSG ratio decreased with predation stress, reaching a minimum after 10 days of exposure (Figure 4A).

Superoxide dismutase 3 (*sod3*) was significantly (*p* < 0.05) elevated after 6 h of predation stress exposure. Radicals peaked after 6 h of predation stress and were significantly (*p* < 0.05) reduced after 10 days of predation stress. The branched-chain amino acids (BCAAs), valine (Val), leucine (Leu), and isoleucine (Ile), increased with increasing predation stress (Figure 4C).

### 3.5 Hemoglobin detection

**FIGURE 5:**
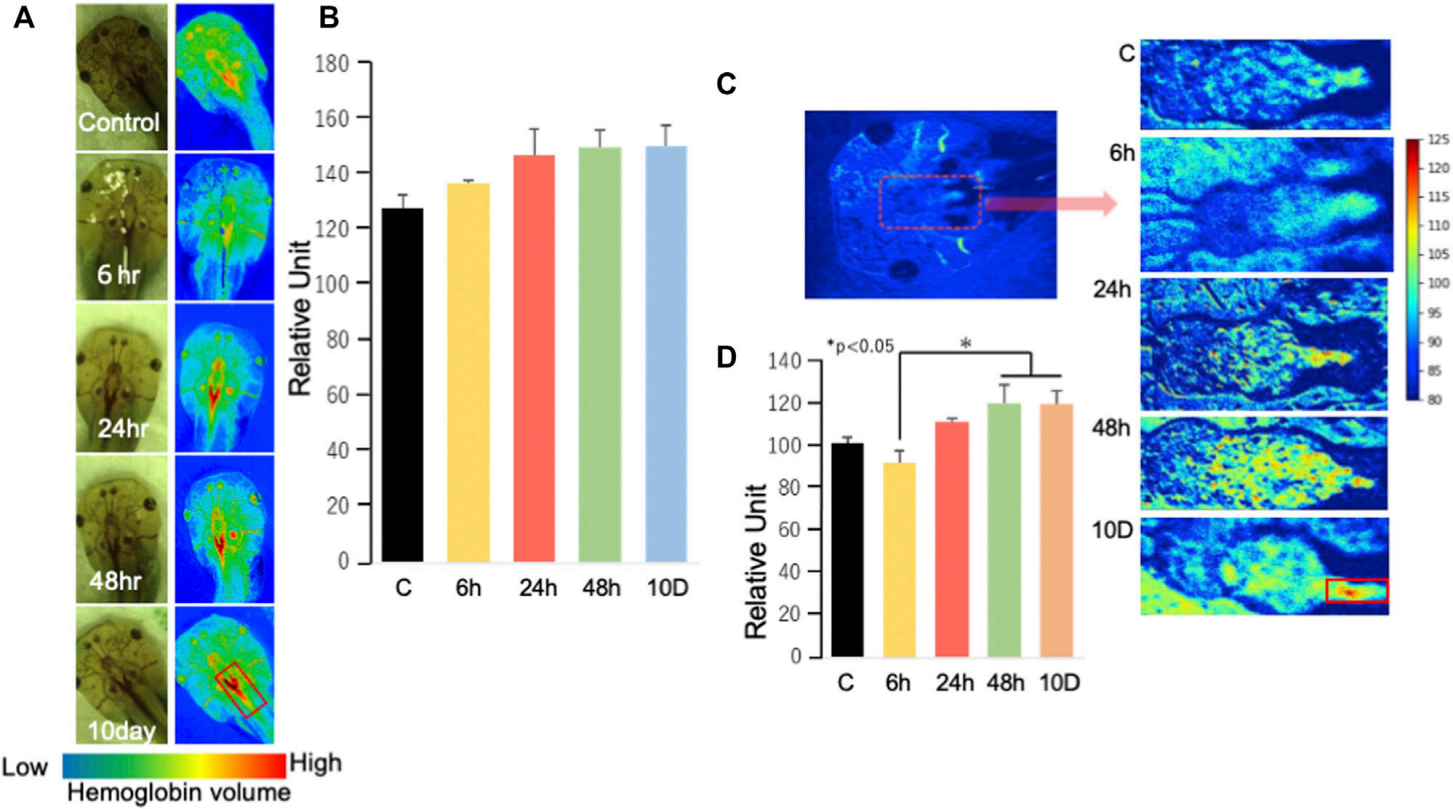
Analysis of hemoglobin using a hyperspectral camera **(A)** Randomly selected tadpoles were analyzed using a hyperspectral camera. The red signal indicates hemoglobin intensity. **(B)** The signal intensity of hemoglobin was determined by averaging the luminance of the area with high intensity (*n* = 2). Data were analyzed using ImageJ (ver1.53e: https://imagej.nih.gov/ij/index.html). **(C)** Oxygen–hemoglobin measurements and C–8D are enlargements of the area surrounded by red dots. **(D)** Oxygen–hemoglobin analysis in the brain was conducted using a similar method to that described in **(B)**. The number of samples used for the control, 6, 24, 48 h, and 10 days groups were four, three, four, three, and two, respectively. Data were analyzed using ImageJ (ver1.53e). Ex 10 days, Ex 48, Ex 24, Ex 6 h, and control are shown as 10 D, 48, 24, 6 h, and C, respectively. *p < 0.05 represented statistical significance based on one-way ANOVA, followed by a Bonferroni *post hoc* test or Dunnett’s T3 test.

Hemoglobin, detected using a hyperspectral camera, became more elevated after 24 h of exposure to predation threat (Figure 5A) than that of the control (Figure 5B). Hemoglobin was observed to bind to oxygen to generate oxyhemoglobin (Figure 5C) (no statistical significance compared with that of the control); however, there was a significant difference between 6-h and the other timepoints (48 h and 10 days) (Figure 5D).

### 3.6 Immunohistochemistry of tadpole brains with a hemoglobin antibody

**FIGURE 6:**
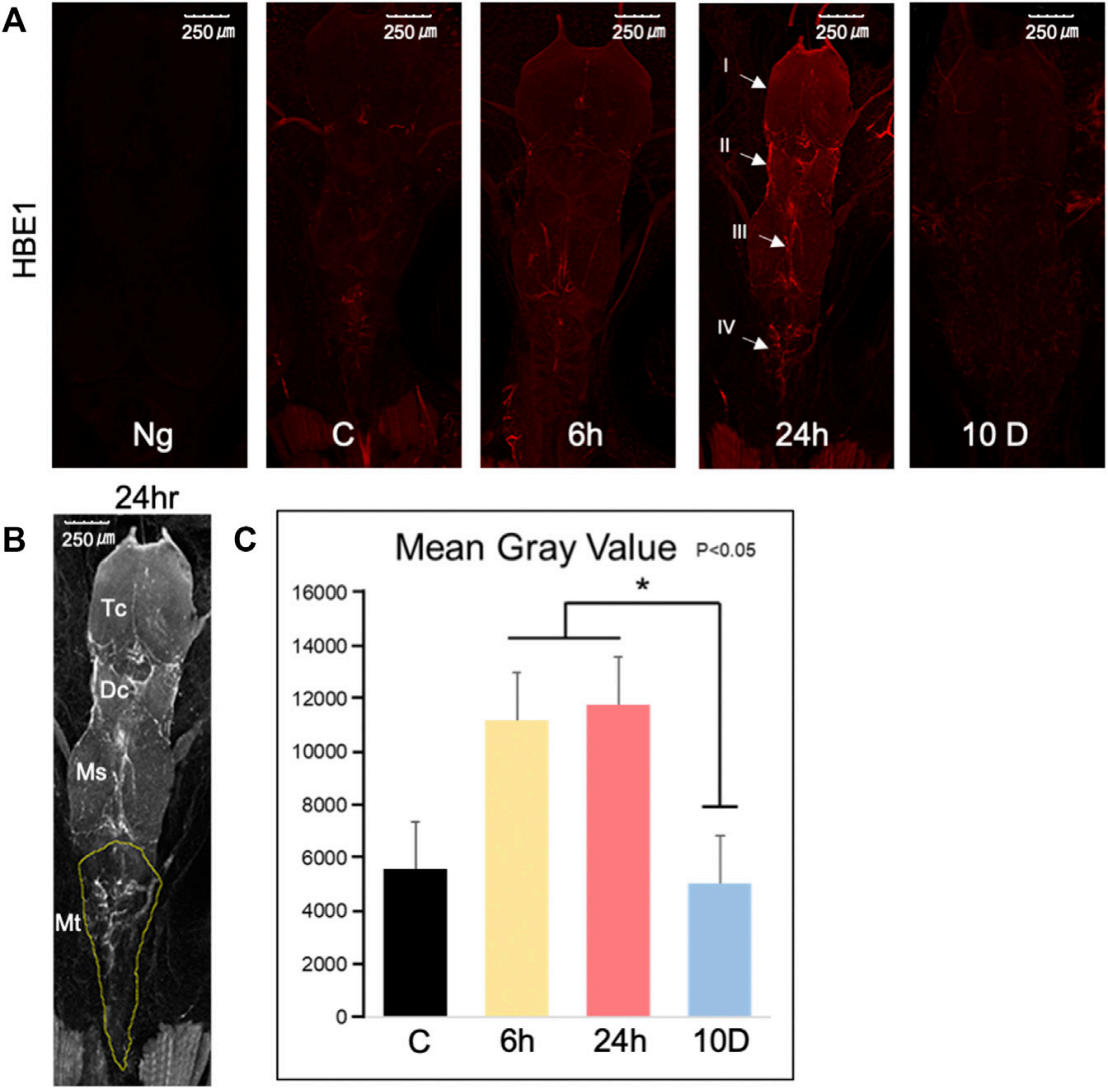
Immunohistochemistry of brains from Xenopus tropicalis tadpoles exposed to predation stress. **(A)** Tadpole brains stained using anti-HBE1 antibody (red). Images were taken using an overlay of constant Z-stack height, under a confocal laser-scanning microscope, with a total of 35 photos in the stack. Negative control (Ng) represents the absence of a primary antibody and the presence of a secondary antibody: control (cont), 6 h (Ex 6 h), 24 h (Ex 24 h), 10 days (Ex 10 days). Parts of the brain shown: (I) around the telencephalon, (II) lateral regions of the diencephalon and mesencephalon; (III) medial zone of the mesencephalon; and (IV) medial zone of the medulla oblongata. **(B)** Measurement of HBE1 intensity in the metencephalon, shown by the dotted yellow line. TC, DC, MS, and MT represent the telencephalon, diencephalon, mesencephalon, and metencephalon, respectively. **(C)** The signal intensity of hemoglobin in MT was determined by averaging the luminance of the area with high intensity . *Xenopus tropicalis* tadpoles were used in this experiment (*n* = 6). Data were analyzed using ImageJ (ver1.53e: https://imagej.nih.gov/ij/index.html). Kolmogorov–Smirnov test showed that the data were normally distributed (*p* = 0.723), whereas Levene’s test showed that the assumed equality of error variance had a significance of 0.05 (*p* = 0.160). *p < 0.05 represented statistical significance based on one-way ANOVA, followed by a *post hoc* comparison using Dunnett’s T3.

The entire brain (Figure 6A) of each tadpole was stained with anti-HBE1 (red), and relatively similar Z-stacks were obtained to compare the signal intensity of antibodies. Notably, no signal was obtained from the negative control (only secondary antibodies). The signal intensity of hemoglobin was higher after 24 h of predation stress than that of the control, as confirmed by Z-stack data (35 slices, Figure 6A). Further, hemoglobin signals were more elevated around the telencephalon (I), lateral regions of the diencephalon, medial zone of the mesencephalon (III), and medial zone of the medulla oblongata (IV; Figure 6B) after 6 and 24 h of predation stress than those of the control (*p* < 0.05; Figure 6C).

### 3.7 IPA of genes expressed in the brain following predation threat

**FIGURE 7:**
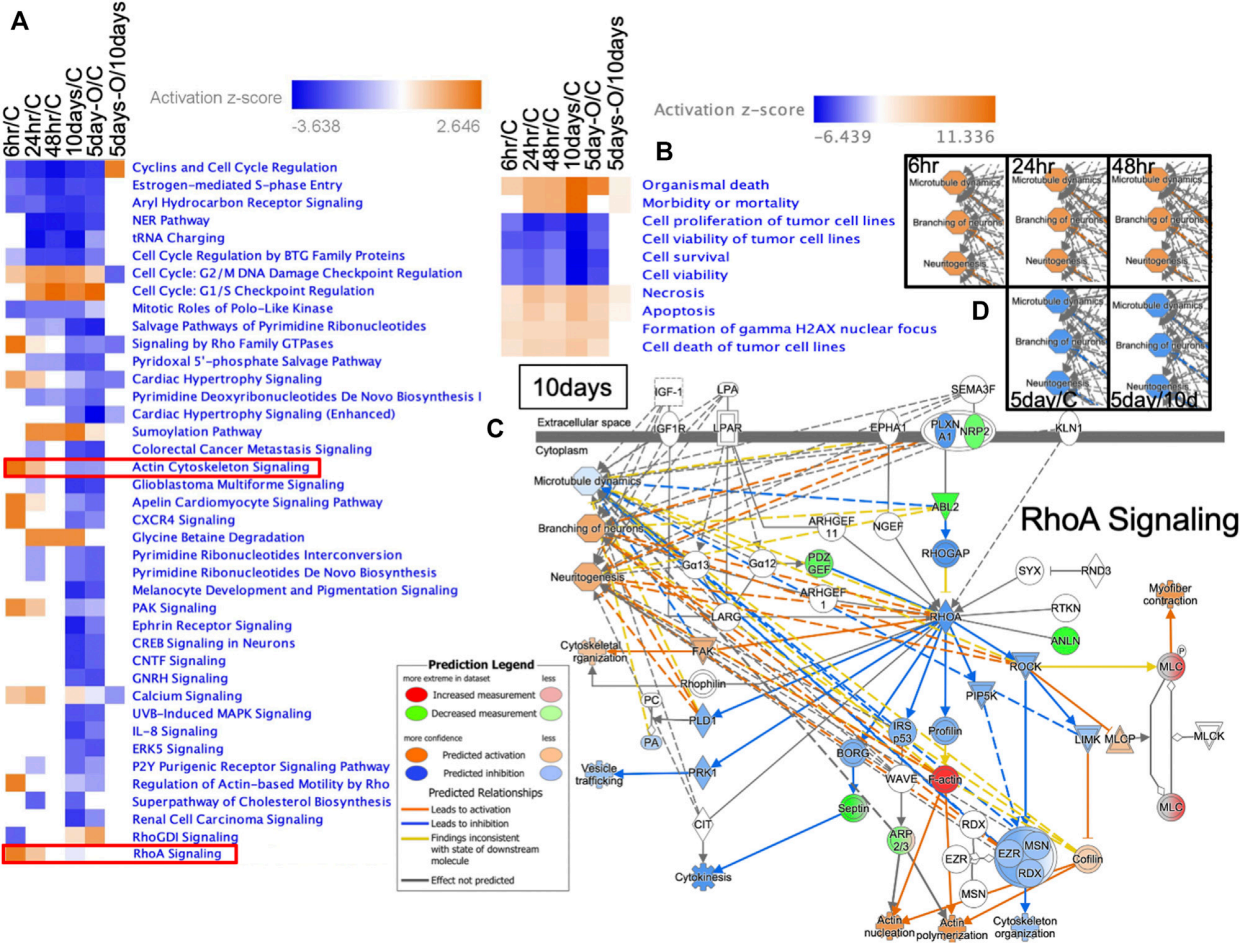
Ingenuity pathway analysis of genes expressed in the brain of *Xenopus tropicalis*. **(A)** Heat map of the signal transduction pathways in each treatment group, where 6 h/C, 24 h/C, 48 h/C, 10 days/C, and 5 days-O/C represent gene expression in the brains of Ex 6, Ex 24, Ex 48 h, Ex 10 days, and Ex 5 days-Out tadpoles divided by that in the control, respectively. Red, blue, and white represent upregulation, downregulation, and no change compared with the control, respectively. **(B)** Disease and function predicted by IPA based on gene expression compared with those in the control and Ex 10 days. Blue and red represent up- and downregulation, respectively. **(C)** Prediction of RhoA signaling based on IPA (Content v.48207413). Microtubule dynamics, branching neurons, and neuritogenesis were predicted according to the comparisons used in **(D)**.

Signal transduction pathways displaying up- or downregulated expression were determined based on z-scores (Figure 7A; all altered signal transduction pathways are shown in Supplementary Table S5). In the Ex 6 h group, six signals were downregulated (including RhoGDI), and ten were upregulated (including RhoA, actin cytoskeleton, CXCR4, and calcium signaling). SUMOylation and glycine betaine degradation pathways were upregulated from 24 h to 10 days after predation stress exposure; however, their respective patterns of upregulation differed significantly (*p* < 0.05; FDR) between the Ex 6 and Ex 48 h groups and the Ex 10 days and Ex 5 days-Out groups (Figure 7A). In the Ex 48 h group, the activity of the various signal transduction pathways did not differ from those in the control, regardless of the downregulation observed at 48 h. G2/M DNA damage checkpoint regulation in the cell cycle was high in all groups.

Disease and function predictions based on all signal transduction cascades are shown in Figure 7B. Organismal death, morbidity, or mortality based on the prediction by IPA analysis was enriched for the Ex 10 days group (all data for disease and function are shown in Supplementary Table S6). The predictions for RhoA signaling are shown in Figure 7C, with these signals being involved in microtubule dynamics, branching of neurons, and neuritogenesis. The microtubule dynamics and neural development of *X. tropicalis* were upregulated from 6 h to ∼48 h of exposure to predation Figure 7D. The top five predictions for Tox function (Supplementary Table S7) indicated increased red blood cells and hematocrit levels across all treatment groups.

## 4 Discussion

In the current study, tadpoles of *X. tropicalis*, belonging to the same genus as *X. laevis*, exhibited no significant morphological changes, such as the exposure duration-dependent tail elongation previously reported for *X. laevis* tadpoles (Mori et al., 2017). However, MANOVA for the first and second principal components of PCA (Figures 1B, C; Supplementary Methods) showed a statistically significant change in morphology after 24 h of exposure relative to the control. In contrast, the third, fourth, and fifth principal components exhibited statistically significant changes among the experimental groups. However, as shown in Figure 1C, there were two groups of experiments in which there was a significant difference in the overall change at sites 4, 9, 10, 12, 13, 14, and 15 in the first component and a common significant difference in the overall change at sites 2, 3, 6, 7, and 8 in the second component. This indicates that there was a certain variation for each component as a whole but it is not clear at this stage what the significance of the change is for any one site of the *X. tropicalis*. However, this difference may indicate that *X. laevis* has a history of encountering salamanders in its larger habitat, while *X. tropicalis* may be a new encounter.

We also investigated physiological conditions in the brain of *X. tropicalis* exposed to predation stress using RNA-seq, real-time PCR, and CE–MS. Ideally, data obtained via these different methods should be in agreement; however, the translational regulation imposed on gene products sometimes leads to differing results on gene expression and metabolite analysis (Ingolia et al., 2009). Thus, in this study, we prioritized the results of metabolite analysis over those of gene expression. Based on RNA-seq, *pdk4* significantly decreased after 6 h of predation stress (Figure 2A), whereas real-time PCR indicated a slightly increasing trend (Figure 3B). Moreover, the protein product of *pdk4* inhibits the conversion of pyruvate to acetyl CoA (Zhang et al., 2014). Only pyruvate levels increased in all stress groups, and statistical analysis showed no significant difference, possibly owing to the small sample size. However, using this approach, Matsui et al. (2017) detected approximately 100 nmol/g of pyruvate in the cerebral cortex and hippocampus of rats via CE-TOFMS, similar to the levels detected in this study. Therefore, a change in pyruvate, a metabolite of glycolysis, can be detected.

Consequently, *pdk4* likely inhibited the entry of glucose into the TCA cycle in the stress groups, converting pyruvate to lactate, which was then utilized by the glycolytic system to generate ATP. Lactic acid also tended to increase from 6 h onwards (Figure 3B), indicating that *pdk4* was expressed after 6 h of predation stress to promote glycolysis. *pdk4* expression was predicted to inhibit the glycolytic system from maintaining glucose levels while promoting lipid and amino acid metabolism via *ß*-oxidation (Zhang et al., 2014). The current study showed that *idh*, the rate-limiting enzyme of the TCA cycle (Stueland et al., 1988), was suppressed in all experimental groups. *fabp* genes, *acsl*, *cpt1*, and *ctp2*, which are involved in *ß*-oxidation, were also suppressed (except in the Ex 48 h group, Figure 3C). Thus, oxidative phosphorylation might have been suppressed in comparison to the control. Of note, the amount of ATP in all experimental groups was approximately the same as that in the control. Thus, energy production via the glycolytic system likely occurred under predation stress. Although the efficiency of ATP production via this system is relatively low, it is ×100 faster than that via oxidative phosphorylation (Stojan and Christopher-Stine, 2015). Therefore, ATP production via glycolysis might still occur as long as glucose is present, to some extent.

In the present study, glycogen storage in the brain was low. It could have depleted after 6 h of predation stress because the expression of *pygm*, which is associated with starch and sucrose metabolism, increased at 6 h (Figure 2B). Furthermore, the significant increase in glucose in the body fluids indicates that glucose was supplied by the liver to support the glycolytic system, accounting for the increase observed at 6 h. Whether *pgm* was involved in subsequent metabolic changes remains unknown, as it was not detected (ND) via RNA-seq. In contrast, glucose-6-phosphatase catalytic subunit 3 (*g6pc3*) was upregulated, indicating that protein-driven glycogenesis was active (see 1.5 Starch and sucrose metabolism via PYGM, G6PC1, PDK4, and G6PC/G6PC2/G6PC3 in the Supplementary Methods). Glycine and serine pathways convert serine to pyruvate, which is then utilized for glycogenesis (Amelio et al., 2014). Furthermore, tyrosine metabolism yields fumarate and acetoacetate (Bateman et al., 2007). Thus, ketone bodies or substrates for glycogenesis are produced without *ß*-oxidation to synthesize fumarate or acetoacetate.

Since free radicals are generated when the TCA cycle is activated, *X. tropicalis* tadpoles may suppress reactive oxygen species (ROS) production by inhibiting the TCA cycle. ROS levels were higher in the Ex 6 h group than in the control (Figure 4A), whereas they were lower in the other experimental groups. This might be attributed to the ROS-scavenging enzyme *sod3* (Nozik-Grayck et al., 2005) (Figure 4A), which protects the vascular system from free radicals. This enzyme was upregulated from 6 h to 10 days of predation stress. The contribution of GSH to ROS removal was low, reaching a minimum at 10 days, whereas GSSG peaked. Accordingly, the GSH/GSSG ratio decreased with stress and was lowest (notably, still >3) at 10 days, reflecting the ability of GSH to remove radicals (Figure 4A).

BCAAs are involved in maple syrup urine disease and are caused by a deficiency of the enzyme branched-chain keto acid dehydrogenase (BCKD) (Abiri et al., 2019). The levels of these BCAAs rose following predation stress. Previous studies showed that the radical-producing metabolites (Fontella et al., 2002) of BCAAs [such as *a*-ketoisocaproic acid (KIC), *a*-ketoisovaleric acid (KIV), and *a*-keto-beta-methylvaleric acid (KMV)] inhibit the mitochondrial respiratory chain (Sgaravatti et al., 2003). However, RNA-seq data from tadpole brains showed 0.93–0.98 and 0.96–0.98 fold changes in *bckdα* and *bckdβ* gene expression, respectively. Furthermore, CE–MS analysis detected KIV, but not KIC or KMV. The metabolism of Leu, Val, and Ile might have been suppressed in *X. tropicalis* tadpoles under predator stress, resulting in increased levels. However, the exact underlying mechanisms need clarifying.

IPA indicated elevated hemoglobin based on the increased levels of red blood cells (Supplementary Table S7) under all predation stress conditions. Real-time PCR showed that the expression of heme synthesis genes (such as 5′-aminolevulinate synthase 2 [*alas2*] and hemoglobin subunit alpha-3 [*hba3*]) increased in all experimental groups compared with that in the control. Specifically, there was a gradual increase in the expression of these genes from 6 to 24 h, and a slower subsequent increase up to 10 days (Figure 2C). Heme synthesis genes *uros* and *ppox* were also upregulated in all experimental groups, indicating that heme synthesis is promoted through gene expression changes. Hyperspectral camera observations showed that hemoglobin tended to increase gradually from 24 h to 10 days of predation stress (Figure 5A), indicating upregulation at the protein level as well. However, since this study aimed to investigate the effects of fear stress on the brain, we did not remove red blood cells from the brain. Therefore, we cannot conclude that hemoglobin was synthesized in brain cells other than red blood cells. Although tissue staining with the HB3 antibody was highest following 24 h of predation stress, HB3 staining produced similar results to that observed with the hyperspectral camera. While hemoglobin produces toxic hydroxyl radicals (Ohyagi et al., 1994), oxyhemoglobin scavenges these, along with peroxynitrite (Boccini and Herold, 2004). In the present study, oxyhemoglobin was specifically upregulated in the midbrain. The midbrain connects the hind and forebrain, with the former controlling the most basic autonomic functions of life, such as breathing, heart rate, digestion, and movement (Nieuwenhuys et al., 1998). Therefore, the increase in oxyhemoglobin levels around the mid and hindbrain indicates a brain function-protective response (Figure 5C).

Brain signal transduction analysis revealed an early response strategy in *X. tropicalis* tadpoles. After 6 h of predation stress, there were six suppressed and ten upregulated signal transduction pathways, respectively (Figure 7A). This result indicated that signal transduction was not inhibited following 48 h of predator exposure but remained active or was at levels similar to those in control specimens. Previous research showed that signal transduction in *X. laevis* is weakly suppressed after 10 days of predator exposure (Mori et al., 2020). The current study identified 33 strongly suppressed signal transduction pathways in *X. tropicalis* tadpoles (Figure 7A). Notably, actin cytoskeleton signaling and RhoA signaling were upregulated after 6 h, whereas RhoGDI was downregulated owing to an increase in the key regulator F-actin. RhoA acts as an on/off switch, whereas RhoGDI acts to stop this switch (DerMardirossian and Bokoch, 2005). Thus, suppression of RhoGDI signaling was predicted to activate the RhoA switch, indicating enhanced turnover within the brain.

Thus, neuritogenesis (Dharmalingam et al., 2009), the formation of cellular protrusions (Ikeda et al., 2001), and neural stem cell differentiation might be enhanced (Lu et al., 2008). Microtubule dynamics in *X. tropicalis* tadpoles were activated from 6 to 48 h of predation stress (Figures 7C,D), with the branching of neurons being predicted to occur following 10 days of predation stress Figure 7D. Thus, the brain network appears to be subjected to various changes from the onset of predator stress. Moreover, glycine betaine degradation and SUMOylation pathways were activated from 24 h to 10 days of predation stress, promoting the antioxidant effects of substances in the brain (Zhang et al., 2016). Abnormal cell division in the brain was predicted to be prevented in all experimental groups owing to the activation of checkpoint regulation (Nozaki et al., 1999). Thus, both physiological changes and brain network restructuring appeared to occur simultaneously in *X. tropicalis* tadpoles.

Collectively, the results of the current study indicate that *X. tropicalis* phenotypic plasticity did not manifest as morphological changes (e.g., extended tail length) in response to the duration of predation stress, but as physiological changes in brain homeostasis and metabolism, including ROS suppression via TCA cycle inhibition and protection of the medulla oblongata through the binding of oxygen and hemoglobin to form oxyhemoglobin, which is essential for proper brain function. This suggests that at least *Xenopus* sp*.* may have a strategy to protect the brain by increasing hemoglobin synthesis during novel predator stress.

## Data Availability

The datasets presented in this study can be found in online repositories. The names of the repository/repositories and accession number(s) can be found below: https://www.ddbj.nig.ac.jp/, PRJDB 14079.
