# Supplementary material for: Novel predator-induced phenotypic plasticity by hemoglobin and physiological changes in the brain of Xenopus tropicalis
Source: Front Physiol. 2023 Jun 6;14:1178869. doi: 10.3389/fphys.2023.1178869 (PMC10279953; doi:10.3389/fphys.2023.1178869)
Supplement: Supplementary file 1 [file Table5.pdf]

Supplementary Table 5

Canonical pathways predicted by IPA. All altered signal transduction pathways used in Fig.7a

© 2000-2019 QIAGEN. All rights reserved.

Canonical Pathways	6hr/cont	24hr/cont	48hr/cont	10days/cont	5day-O/cont	5day-O/10days
Cyclins and Cell Cycle Regulation	-2.449	-3.051	-3.464	-3.051	-2.828	2.236
Estrogen-mediated S-phase Entry	-2	-2.53	-3	-2.53	-2.646	N/A
Aryl Hydrocarbon Receptor Signaling	-2.236	-2.111	-2.828	-2.887	-2.53	N/A
NER Pathway	N/A	-3.317	-3.317	-3	-2.646	N/A
tRNA Charging	N/A	-3.464	-2.828	-3.464	-1.342	N/A
Cell Cycle Regulation by BTG Family Proteins	-1	-2.646	-2.646	-2.828	-1.89	N/A
Cell Cycle: G2/M DNA Damage Checkpoint Regulation	1	1.667	1.897	1.732	0.816	-2.236
Cell Cycle: G1/S Checkpoint Regulation	N/A	1.897	2.646	2.111	2.646	N/A
Mitotic Roles of Polo-Like Kinase	-2.236	-1.897	-1.897	-1.897	-0.816	N/A
Salvage Pathways of Pyrimidine Ribonucleotides	N/A	-1.414	-1.134	-2.84	-3.162	N/A
Signaling by Rho Family GTPases	2.449	0.378	N/A	-1.886	-1.667	-2
Pyridoxal 5'-phosphate Salvage Pathway	N/A	-1.342	-1.342	-2.53	-2.449	N/A
Cardiac Hypertrophy Signaling	1.633	0.816	N/A	-0.943	-1.941	-1.89
Pyrimidine Deoxyribonucleotides De Novo Biosynthesis I	N/A	-1.633	-1.342	-1.89	-2.236	N/A
Cardiac Hypertrophy Signaling (Enhanced)	0	0	N/A	-2.043	-3.638	-1.265
Sumoylation Pathway	N/A	2	2	2.309	0.447	N/A
Colorectal Cancer Metastasis Signaling	N/A	-1.342	N/A	-2.496	-2.714	N/A
Actin Cytoskeleton Signaling	2.449	1.134	N/A	-1.5	-1.414	N/A
Glioblastoma Multiforme Signaling	N/A	-1	N/A	-2.828	-2.646	N/A
Apelin Cardiomyocyte Signaling Pathway	2.236	0.447	N/A	-1.508	-2.121	N/A
CXCR4 Signaling	2.236	0	N/A	-2.138	-1.667	N/A
Glycine Betaine Degradation	N/A	2	2	2	N/A	N/A
Pyrimidine Ribonucleotides De Novo Biosynthesis	N/A	-1.342	N/A	-1.89	-2.236	N/A
Pyrimidine Ribonucleotides Interconversion	N/A	-1.342	N/A	-1.89	-2.236	N/A
Melanocyte Development and Pigmentation Signaling	N/A	N/A	N/A	-3	-2.449	N/A
PAK Signaling	2	1	N/A	-1.414	-1	N/A
Ephrin Receptor Signaling	N/A	N/A	N/A	-3.207	-1.89	N/A
CREB Signaling in Neurons	N/A	N/A	N/A	-2.828	-2.236	N/A
CNTF Signaling	N/A	N/A	N/A	-2.828	-2.236	N/A

GNRH Signaling	N/A	N/A	N/A	-2.309	-2.53	N/A
Calcium Signaling	1	1.414	N/A	0.535	-0.333	-1.508
UVB-Induced MAPK Signaling	N/A	N/A	N/A	-2.449	-2.236	N/A
IL-8 Signaling	N/A	0	N/A	-2.714	-1.897	N/A
ERK5 Signaling	N/A	N/A	N/A	-2.121	-2.449	N/A
P2Y Purigenic Receptor Signaling Pathway	N/A	-1	N/A	-2.121	-1.414	N/A
Regulation of Actin-based Motility by Rho	2.236	N/A	N/A	-0.905	-1.342	N/A
Superpathway of Cholesterol Biosynthesis	N/A	-2.236	N/A	-2.236	N/A	N/A
Renal Cell Carcinoma Signaling	N/A	N/A	N/A	-2.828	-1.633	N/A
RhoGDI Signaling	-2.236	N/A	N/A	0.577	1.633	N/A
NGF Signaling	N/A	N/A	N/A	-2.53	-1.89	N/A
Nitric Oxide Signaling in the Cardiovascular System	N/A	-1.342	N/A	-1.414	-1.633	N/A
Rac Signaling	N/A	N/A	N/A	-2.714	-1.633	N/A
Melanoma Signaling	N/A	N/A	N/A	-2.236	-2	N/A
Growth Hormone Signaling	N/A	-0.447	N/A	-1.897	-1.89	N/A
Endocannabinoid Developing Neuron Pathway	N/A	N/A	N/A	-2.53	-1.667	N/A
Angiopoietin Signaling	N/A	N/A	N/A	-2.121	-2	N/A
G Beta Gamma Signaling	N/A	0	N/A	-1.667	-2.449	N/A
FGF Signaling	N/A	N/A	N/A	-2.449	-1.633	N/A
Thrombin Signaling	N/A	N/A	N/A	-2.121	-1.89	N/A
FLT3 Signaling in Hematopoietic Progenitor Cells	N/A	N/A	N/A	-2.333	-1.633	N/A
Agrin Interactions at Neuromuscular Junction	N/A	N/A	N/A	-1.633	-2.236	N/A
Role of BRCA1 in DNA Damage Response	-1.633	-0.832	0	-1.213	0	N/A
Cardiac β-adrenergic Signaling	N/A	0	N/A	-1.667	-2	0
3-phosphoinositide Degradation	N/A	N/A	N/A	-1.941	-1.667	N/A
RhoA Signaling	2.236	1	N/A	-0.333	N/A	N/A
Fcγ Receptor-mediated Phagocytosis in Macrophages and Monocyte	N/A	N/A	N/A	-1.265	-2.121	N/A
eNOS Signaling	N/A	N/A	N/A	-1.414	-1.89	N/A
Relaxin Signaling	N/A	N/A	N/A	-1.667	-1.633	N/A
Apelin Adipocyte Signaling Pathway	N/A	N/A	N/A	-1.633	-1.633	N/A
Leukocyte Extravasation Signaling	N/A	0	N/A	-0.905	-2.121	N/A
HGF Signaling	N/A	N/A	N/A	-2.121	-0.816	N/A
Ovarian Cancer Signaling	N/A	N/A	N/A	-1.134	-1.633	N/A
Insulin Receptor Signaling	N/A	-0.447	N/A	-1.265	-1	N/A

Endocannabinoid Neuronal Synapse Pathway	N/A	N/A	N/A	−1.291	−1.414	N/A
Small Cell Lung Cancer Signaling	N/A	N/A	N/A	−2.236	N/A	N/A
Antiproliferative Role of Somatostatin Receptor 2	N/A	N/A	N/A	−2.236	N/A	N/A
Type II Diabetes Mellitus Signaling	N/A	N/A	N/A	−0.816	−1.342	N/A
Role of CHK Proteins in Cell Cycle Checkpoint Control	N/A	0.707	0.707	0.333	0.378	N/A
Glioma Signaling	N/A	N/A	N/A	−2	N/A	N/A
AMPK Signaling	N/A	N/A	N/A	−0.775	−1.155	0
p53 Signaling	N/A	0.447	0	0.302	1	N/A
ATM Signaling	0	0	0.333	−0.775	−0.632	N/A
Sirtuin Signaling Pathway	0	−0.277	0	−1.043	−0.229	N/A
Pancreatic Adenocarcinoma Signaling	N/A	0.447	N/A	−1	0	N/A
Acute Phase Response Signaling	N/A	1	N/A	−0.333	0	N/A
Notch Signaling	N/A	N/A	N/A	−1	N/A	N/A
Fc γ RIIB Signaling in B Lymphocytes	N/A	N/A	N/A	−1	N/A	N/A
Inhibition of Matrix Metalloproteases	N/A	N/A	N/A	0	1	N/A
Gluconeogenesis I	N/A	N/A	N/A	1	N/A	N/A
Superpathway of Methionine Degradation	N/A	0	N/A	−0.447	N/A	N/A
PI3K/AKT Signaling	N/A	N/A	N/A	−0.378	0	N/A
