# Supplementary material for: Novel predator-induced phenotypic plasticity by hemoglobin and physiological changes in the brain of Xenopus tropicalis
Source: Front Physiol. 2023 Jun 6;14:1178869. doi: 10.3389/fphys.2023.1178869 (PMC10279953; doi:10.3389/fphys.2023.1178869)
Supplement: Supplementary file 2 [file Table4.pdf]

Supplementary Table 4
MANOVA of PCA

Between-subject factor

		N
Ex group	10day	60
	24h	44
	48h	28
	5day out	27
	Cont	30

Descriptive statistics

Ex group		Mean	Std. Deviation	N
First Component	10day	-.1369	.90535	60
	24h	-.3070	.93239	44
	48h	-.1163	1.24215	28
	5day out	.4514	.85703	27
	Cont	.4264	.92656	30
	Total	.0000	1.00000	189
Second Component	10day	-.0195	.96548	60
	24h	-.5955	.95620	44
	48h	.3688	.90268	28
	5day out	.1556	.99037	27
	Cont	.4279	.84291	30
	Total	.0000	1.00000	189
Third Component	10day	-.3352	.74352	60
	24h	-.2140	.61640	44
	48h	1.3100	1.46321	28
	5day out	-.1582	.75513	27
	Cont	-.0960	.54622	30
	Total	.0000	1.00000	189
Fourth Component	10day	-.4716	.96566	60
	24h	.5343	.72462	44
	48h	.1964	.97847	28
	5day out	-.2876	1.00645	27
	Cont	.2351	.97099	30
	Total	.0000	1.00000	189
Fifth Component	10day	-.2848	.83192	60
	24h	.2227	1.16206	44
	48h	-.3241	.83986	28
	5day out	.0350	.91866	27
	Cont	.5140	1.01987	30
	Total	.0000	1.00000	189

Multivariate tests (a)

Effect		Value	F	Hypothesis df	Error df	Sig.	Partial Eta Squared
Intercept	Pillai's Trace	.028	1.030[b]	5.000	180.000	.402	.028
	Wilkins' Lambda	.972	1.030[b]	5.000	180.000	.402	.028
	Hotelling's Trace	.029	1.030[b]	5.000	180.000	.402	.028
	Roy's Largest Root	.029	1.030[b]	5.000	180.000	.402	.028
Ex group	Pillai's Trace	.791	9.027	20.000	732.000	.000	.198
	Wilkins' Lambda	.393	9.710	20.000	597.942	.000	.208
	Hotelling's Trace	1.115	9.951	20.000	714.000	.000	.218
	Roy's Largest Root	.570	20.850[c]	5.000	183.000	.000	.363

a: Computed using alpha = 0.05

b: Exact static

c: The statistic is an upper bound of F that yields a lower bound of the significance level.

Test for homogeneity of variances

	Levene Statistic	df 1	df 2	Sig.
First Component	2.490	4	184	.045
Second Component	.137	4	184	.969
Third Component	12.985	4	184	.000
Fourth Component	1.542	4	184	.192
Fifth Component	.699	4	184	.594

Tests of between-subject effects

Source Variable	Dependent	Type III Sum of Squares	df	Mean Square	F	Sig.	Partial Eta Squared
Corrected Model	First Component	16.605[a]	4	4.151	4.457	.002	.088
	Second Component	25.581[b]	4	6.395	7.245	.000	.136
	Third Component	57.761[c]	4	14.440	20.401	.000	.307
	Fourth Component	30.877[d]	4	7.719	9.040	.000	.164
	Fifth Component	17.949[e]	4	4.487	4.855	.001	.095
Intercept	First Component	.693	1	.693	.744	.389	.004
	Second Component	.783	1	.783	.887	.348	.005
	Third Component	1.764	1	1.764	2.492	.116	.013
	Fourth Component	.293	1	.293	.344	.558	.002
	Fifth Component	.182	1	.182	.197	.658	.001
Exgroup	First Component	16.605	4	4.151	4.457	.002	.088
	Second Component	25.581	4	6.395	7.245	.000	.136
	Third Component	57.761	4	14.440	20.401	.000	.307
	Fourth Component	30.877	4	7.719	9.040	.000	.164
	Fifth Component	17.949	4	4.487	4.855	.001	.095
Error	First Component	171.395	184	.931			
	Second Component	162.419	184	.883			
	Third Component	130.239	184	.708			
	Fourth Component	157.123	184	.854			
	Fifth Component	170.051	184	.924			
Total	First Component	188.000	189				
	Second Component	188.000	189				
	Third Component	188.000	189				
	Fourth Component	188.000	189				
	Fifth Component	188.000	189				
Corrected Total	First Component	188.000	188				
	Second Component	188.000	188				
	Third Component	188.000	188				
	Fourth Component	188.000	188				
	Fifth Component	188.000	188				

a: R Squared = .088 (Adjusted R Squared = .069)
b: R Squared = .136 (Adjusted R Squared = .117)
c: R Squared = .307 (Adjusted R Squared = .292)
d: R Squared = .164 (Adjusted R Squared = .146)
e: R Squared = .095 (Adjusted R Squared = .076)

Multiple Comparisons

Dependent Variable	(I) Ex group	(J) Ex group		Mean Difference (I-J)	Std. Error	Sig.	95% Confidence Interval Lower Bound	Upper Bound
First Component	Dunnett T3	10day	24h	.1701	.18281	.986	-.3537	.6939
			48h	-.0206	.26223	1.000	-.7938	.7526
			5day out	-.5883	.20215	.051	-1.1774	.0008
			Cont	-.5632	.20562	.077	-1.1607	.0342
		24h	10day	-.1701	.18281	.986	-.6939	.3537
			48h	-.1907	.27361	.998	-.9928	.6113
			5day out	-.7584*	.21671	.009	-1.3874	-.1294
			Cont	-.7334*	.21994	.014	-1.3703	-.0965
		48h	10day	.0206	.26223	1.000	-.7526	.7938
			24h	.1907	.27361	.998	-.6113	.9928
			5day out	-.5677	.28689	.407	-1.4071	.2717
			Cont	-.5427	.28935	.479	-1.3879	.3026
		5day out	10day	.5883	.20215	.051	-.0008	1.1774
			24h	.7584*	.21671	.009	.1294	1.3874
			48h	.5677	.28689	.407	-.2717	1.4071
			Cont	.0250	.23626	1.000	-.6624	.7125
		Cont	10day	.5632	.20562	.077	-.0342	1.1607
			24h	.7334*	.21994	.014	.0965	1.3703
			48h	.5427	.28935	.479	-.3026	1.3879
			5day out	-.0250	.23626	1.000	-.7125	.6624
Second Component	Bonferroni	10day	24h	.5760*	.18648	.023	.0462	1.1058
			48h	-.3883	.21503	.726	-.9993	.2227
			5day out	-.1751	.21773	1.000	-.7937	.4436
			Cont	-.4474	.21008	.345	-1.0443	.1495
		24h	10day	-.5760*	.18648	.023	-1.1058	-.0462
			48h	-.9643*	.22713	.000	-1.6096	-.3190
			5day out	-.7511*	.22968	.013	-1.4037	-.0985
			Cont	-1.0234*	.22245	.000	-1.6555	-.3913
		48h	10day	.3883	.21503	.726	-.2227	.9993
			24h	.9643*	.22713	.000	.3190	1.6096
			5day out	.2132	.25341	1.000	-.5068	.9333
			Cont	-.0591	.24688	1.000	-.7605	.6424
		5day out	10day	.1751	.21773	1.000	-.4436	.7937
			24h	.7511*	.22968	.013	.0985	1.4037
			48h	-.2132	.25341	1.000	-.9333	.5068
			Cont	-.2723	.24923	1.000	-.9805	.4358
		Cont	10day	.4474	.21008	.345	-.1495	1.0443
			24h	1.0234*	.22245	.000	.3913	1.6555
			48h	.0591	.24688	1.000	-.6424	.7605
			5day out	.2723	.24923	1.000	-.4358	.9805
Third Component	Dunnett T3	10day	24h	-.1213	.13360	.988	-.5032	.2607
			48h	-1.6452*	.29271	.000	-2.5180	-.7725
			5day out	-.1770	.17416	.972	-.6859	.3319
			Cont	-.2393	.13842	.588	-.6378	.1592
		24h	10day	.1213	.13360	.988	-.2607	.5032
			48h	-1.5240*	.29172	.000	-2.3946	-.6533
			5day out	-.0558	.17250	1.000	-.5610	.4495
			Cont	-.1180	.13631	.991	-.5119	.2758
		48h	10day	1.6452*	.29271	.000	.7725	2.5180

			24h	1.5240*	.29172	.000	.6533	2.3946
			5day out	1.4682*	.31238	.000	.5470	2.3895
			Cont	1.4060*	.29395	.000	.5299	2.2820
		5day out	10day	.1770	.17416	.972	-.3319	.6859
			24h	.0558	.17250	1.000	-.4495	.5610
			48h	-1.4682*	.31238	.000	-2.3895	-.5470
			Cont	-.0623	.17625	1.000	-.5785	.4540
		Cont	10day	.2393	.13842	.588	-.1592	.6378
			24h	.1180	.13631	.991	-.2758	.5119
			48h	-1.4060*	.29395	.000	-2.2820	-.5299
			5day out	.0623	.17625	1.000	-.4540	.5785
Fourth Component	Bonferroni	10day	24h	-1.0059*	.18341	.000	-1.5270	-.4848
			48h	-.6680*	.21149	.019	-1.2689	-.0671
			5day out	-.1840	.21415	1.000	-.7925	.4244
			Cont	-.7067*	.20663	.008	-1.2938	-.1197
		24h	10day	1.0059*	.18341	.000	.4848	1.5270
			48h	.3379	.22339	1.000	-.2968	.9726
			5day out	.8219*	.22591	.004	.1800	1.4637
			Cont	.2992	.21880	1.000	-.3225	.9208
		48h	10day	.6680*	.21149	.019	.0671	1.2689
			24h	-.3379	.22339	1.000	-.9726	.2968
			5day out	.4840	.24925	.537	-.2242	1.1921
			Cont	-.0388	.24282	1.000	-.7287	.6512
		5day out	10day	.1840	.21415	1.000	-.4244	.7925
			24h	-.8219*	.22591	.004	-1.4637	-.1800
			48h	-.4840	.24925	.537	-1.1921	.2242
			Cont	-.5227	.24514	.343	-1.2192	.1738
		Cont	10day	.7067*	.20663	.008	.1197	1.2938
			24h	-.2992	.21880	1.000	-.9208	.3225
			48h	.0388	.24282	1.000	-.6512	.7287
			5day out	.5227	.24514	.343	-.1738	1.2192
Fifth Component	Bonferroni	10day	24h	-.5075	.19081	.085	-1.0496	.0346
			48h	.0392	.22002	1.000	-.5859	.6644
			5day out	-.3199	.22278	1.000	-.9529	.3131
			Cont	-.7989*	.21496	.003	-1.4096	-.1881
		24h	10day	.5075	.19081	.085	-.0346	1.0496
			48h	.5467	.23240	.197	-.1136	1.2070
			5day out	.1876	.23502	1.000	-.4801	.8554
			Cont	-.2914	.22762	1.000	-.9381	.3554
		48h	10day	-.0392	.22002	1.000	-.6644	.5859
			24h	-.5467	.23240	.197	-1.2070	.1136
			5day out	-.3591	.25930	1.000	-1.0958	.3777
			Cont	-.8381*	.25261	.011	-1.5558	-.1203
		5day out	10day	.3199	.22278	1.000	-.3131	.9529
			24h	-.1876	.23502	1.000	-.8554	.4801
			48h	.3591	.25930	1.000	-.3777	1.0958
			Cont	-.4790	.25502	.619	-1.2036	.2456
		Cont	10day	.7989*	.21496	.003	.1881	1.4096
			24h	.2914	.22762	1.000	-.3554	.9381
			48h	.8381*	.25261	.011	.1203	1.5558
			5day out	.4790	.25502	.619	-.2456	1.2036

* indicates significance at 0.05
