# Supplementary material for: Novel predator-induced phenotypic plasticity by hemoglobin and physiological changes in the brain of Xenopus tropicalis
Source: Front Physiol. 2023 Jun 6;14:1178869. doi: 10.3389/fphys.2023.1178869 (PMC10279953; doi:10.3389/fphys.2023.1178869)
Supplement: Supplementary file 3 [file Table1.pdf]

Supplementary Table 1

Body part measurements. The fifteen body parameters of tadpoles measured for the principal component analysis described in Fig.1b.

Experimenta l Group	Part of body No														
	No1	No2	No3	No4	No5	No6	No7	No8	No9	No10	No11	No12	No13	No14	No15
Control	0.339	7.24927	5.16093	5.47446	1.37636	2.03045	0.83671	2.94533	6.29180	2.86216	0.26331	3.52568	2.77272	0.22418	2.68476
Control	0.352	7.66424	5.32066	6.17467	1.14544	2.55637	0.78706	2.85865	6.96750	2.65757	0.29838	3.77024	2.67504	0.29609	3.22336
Control	0.258	8.32717	4.07052	7.69309	1.80839	1.93409	0.73238	2.60335	7.11470	3.40063	0.44172	4.12479	3.10129	0.52159	3.61804
Control	0.336	7.48400	5.11390	5.79715	1.48648	2.57245	0.83510	3.20647	6.53930	2.89425	0.26587	4.27608	3.03938	0.33476	2.87983
Control	0.343	6.88496	5.59174	5.96708	1.55615	2.17727	0.77413	2.91580	7.04340	3.19883	0.27714	4.02826	3.17152	0.30985	3.11135
Control	0.230	7.15523	3.57616	5.88523	0.88482	1.60995	0.78716	2.21185	7.87510	3.13858	0.38654	4.59867	3.00463	0.50146	3.83696
Control	0.245	8.05400	3.54285	6.22070	1.52733	1.94830	0.68409	2.32367	9.21570	3.48924	0.35600	4.92469	3.62087	0.42492	4.00212
Control	0.196	7.17870	3.85044	6.26846	1.38508	1.81981	0.69862	2.79356	6.80190	3.13584	0.30435	4.57496	2.95662	0.35052	3.45002
Control	0.265	6.91275	6.56275	6.67136	0.92348	2.32445	1.05293	3.28498	9.11400	3.19739	0.37148	4.61025	3.43868	0.42822	3.71294
Control	0.272	8.09743	3.70956	7.56264	1.47941	2.04351	0.75676	2.79998	8.81040	3.65244	0.34652	5.13044	3.60974	0.47646	3.78661
Control	0.195	6.24534	5.96976	5.63019	0.68696	2.12642	0.79096	2.63681	8.43320	2.85342	0.39968	3.97056	2.86064	0.35971	3.24302
Control	0.188	6.47150	5.33660	6.27854	1.31940	1.97950	0.75245	2.44471	8.27150	2.82963	0.31692	3.92559	2.75796	0.24309	3.00159
Control	0.216	6.59558	5.41812	5.38600	1.47313	1.96709	0.95580	3.14848	6.99220	2.84509	0.20000	4.16961	3.10891	0.43268	3.06978
Control	0.266	6.58480	5.85800	6.17828	1.61244	1.92747	0.98122	3.19136	7.82100	3.20624	0.38417	4.56088	3.05472	0.41246	3.31228
Control	0.239	6.33528	4.62100	6.79488	1.18113	2.00196	0.87692	2.63777	7.93940	2.81429	0.33304	4.52375	3.06740	0.37467	3.52679
Control	0.259	7.16635	4.40885	6.84740	1.60320	1.97320	0.75169	2.41749	7.74240	2.87983	0.30678	4.59546	3.04279	0.42355	3.67046
Control	0.293	7.67520	4.00680	6.95709	1.26300	2.04062	0.85706	2.69362	7.42800	2.83608	0.39529	4.30863	2.79508	0.46583	3.30192
Control	0.253	5.64027	6.94363	5.21815	1.47857	2.18962	0.82797	2.69173	6.22750	3.02539	0.34615	4.38439	2.84739	0.38654	2.97691
Control	0.155	6.29532	5.28448	5.55494	1.23944	1.89932	0.85008	2.57272	7.07590	2.91886	0.34590	4.39095	2.77303	0.38626	3.20311
Control	0.285	6.43458	6.30382	5.39485	1.10739	2.36577	0.85812	2.30466	6.78240	3.01611	0.31715	4.34632	3.11590	0.32862	3.27001
Control	0.297	6.19128	4.23882	5.24357	0.97183	1.99359	0.61447	2.28944	6.90550	3.03714	0.29120	3.61572	3.02189	0.48664	3.03592
Control	0.270	6.81667	5.75533	7.50168	1.66130	2.08663	0.71819	2.79375	6.97670	3.37409	0.29120	5.40681	3.05720	0.56568	4.49240
Control	0.165	6.39597	6.47093	5.59612	1.66712	2.27753	0.89982	3.17678	7.65300	3.12171	0.25328	3.71175	2.83273	0.49527	4.24103
Control	0.221	6.40612	3.84604	5.61716	1.44940	2.25172	0.51450	3.07213	6.89200	2.69956	0.40049	4.79202	2.67245	0.40049	2.96278
Control	0.235	6.41942	4.73043	5.72046	1.49604	2.13363	0.96832	2.94330	6.96840	3.41083	0.35477	4.66221	3.50717	0.34240	3.47438
Control	0.310	6.30910	5.91320	5.77478	1.22376	2.09728	0.73309	2.76069	7.29650	2.98904	0.28284	3.97836	3.04972	0.30464	3.21448
Control	0.206	7.96978	3.87182	6.83344	1.14912	1.96702	0.63234	2.49038	7.45580	3.35166	0.36326	5.44071	3.31518	0.45407	3.79494
Control	0.215	6.76012	4.66476	6.74592	1.88000	1.87027	0.79341	2.47298	6.68150	2.99388	0.29097	4.45708	3.08312	0.25280	3.40352
Control	0.282	8.01004	2.32946	5.97128	1.45604	1.95896	0.67604	3.07633	6.68100	2.57539	0.23077	3.58586	2.68450	0.26923	2.55175
Control	0.332	6.06567	3.41197	6.40735	0.93049	1.75983	0.92372	2.92003	6.89840	3.03135	0.31974	3.89900	2.99496	0.32222	3.10648

Ex 10days	0.175	8.20833	2.38677	7.21119	1.41667	2.20192	0.64045	2.82869	6.78690	3.41504	0.35971	4.81664	3.38372	0.36851	3.66192
Ex 10days	0.288	5.78529	6.28701	5.60258	1.69516	2.33567	0.78481	2.68330	6.32100	2.84676	0.38383	4.12484	2.89800	0.31706	3.29120
Ex 10days	0.213	7.04672	3.62146	5.78885	1.90072	1.85182	0.65469	2.61886	6.62200	2.65144	0.30307	4.31478	2.55744	0.29437	3.01459
Ex 10days	0.238	5.73746	5.55344	5.74171	1.53269	1.94780	0.73792	2.72430	6.89060	2.72113	0.28284	4.18433	2.74875	0.32984	2.73004
Ex 10days	0.234	7.24708	4.40012	6.72500	1.53734	2.15664	0.77946	2.67623	5.78030	2.89456	0.31240	3.36990	2.83885	0.43268	2.46251
Ex 10days	0.230	6.19740	3.54108	5.71773	1.15145	2.37165	0.66168	2.50925	6.16050	3.26764	0.35818	4.73944	3.29695	0.53727	3.59565
Ex 10days	0.179	6.92324	3.28996	6.59880	1.85300	2.24451	0.58922	2.10130	7.97220	3.64582	0.34358	5.33068	3.70867	0.43500	4.36878
Ex 10days	0.210	6.48713	3.94856	6.16076	1.13037	2.40166	0.70501	2.58042	7.50430	2.95052	0.30333	3.75448	2.87136	0.30333	3.12436
Ex 10days	0.125	6.47069	2.58721	6.00612	1.37442	1.84775	0.66724	2.22720	7.39200	2.84676	0.40792	4.70608	3.13740	0.44720	3.29120
Ex 10days	0.198	6.98937	3.83130	5.38576	1.35244	2.07456	0.63243	2.65627	7.35460	3.13048	0.56000	4.02492	3.12360	0.51315	3.00532
Ex 10days	0.224	7.42000	3.82709	5.98202	1.54167	2.29064	0.54399	2.62913	6.79280	2.93242	0.51538	3.95331	2.95854	0.61942	3.21239
Ex 10days	0.174	5.32386	5.87904	4.70392	1.49989	1.97312	0.64428	2.25535	6.56230	2.77350	0.31692	3.65081	2.87204	0.31692	3.05692
Ex 10days	0.166	5.78473	4.55707	5.63772	1.77300	1.93459	0.62500	2.10936	6.46480	3.12031	0.19936	4.75988	3.32051	0.35885	3.23808
Ex 10days	0.140	6.02376	4.18064	4.98394	0.85610	2.08079	0.60665	2.28413	6.00070	2.67404	0.24296	2.84084	2.69696	0.26679	2.31204
Ex 10days	0.205	5.28217	6.31613	5.60869	1.88238	2.21654	0.69816	2.82027	6.06810	2.98027	0.34149	4.09692	2.99796	0.34149	3.42813
Ex 10days	0.217	6.27877	5.10543	5.90296	1.55431	2.05847	0.51243	2.39664	5.93670	3.01911	0.28000	4.36367	3.06923	0.32248	3.47054
Ex 10days	0.244	6.54848	3.66412	4.86099	0.64620	1.87052	0.77922	2.80547	6.24060	2.90816	0.20380	3.39253	3.27164	0.24313	2.68765
Ex 10days	0.248	6.30213	5.93367	5.45600	1.30723	2.33043	0.81032	2.62862	5.89660	2.58333	0.23379	3.66692	2.58467	0.27176	2.87529
Ex 10days	0.169	6.03146	4.37634	5.22174	0.68477	2.19126	0.62808	2.35388	6.18910	2.91056	0.25612	2.60620	2.96456	0.31240	2.59821
Ex 10days	0.186	5.63178	6.17542	5.91667	1.70457	1.79588	0.77549	2.85970	6.03500	3.27822	0.16972	3.91412	3.34212	0.23324	2.79759
Ex 10days	0.155	7.25970	3.55248	6.08436	1.14642	1.85414	0.75402	2.11119	6.36210	3.05788	0.27926	3.79508	2.92617	0.34328	3.78433
Ex 10days	0.146	5.92637	3.63037	4.75716	1.62412	1.75682	0.72238	2.59963	6.32650	3.00779	0.38462	4.75750	2.92153	0.42308	3.68257
Ex 10days	0.148	7.01350	2.92340	6.37438	1.24304	1.95810	0.67362	1.81339	7.65320	3.29475	0.29097	4.10861	3.28375	0.37918	3.04086
Ex 10days	0.185	6.88117	4.03583	5.26930	1.52670	1.78095	0.77132	2.19608	7.48540	2.56586	0.24309	2.98993	2.61374	0.24309	2.40755
Ex 10days	0.212	7.35228	3.13740	6.17004	1.78000	2.11295	0.72815	2.24243	6.55910	3.33750	0.24000	4.21575	3.25425	0.24000	3.50892
Ex 10days	0.192	5.70145	5.51865	4.47123	1.29487	2.45747	0.61759	2.51881	6.82120	2.66719	0.23131	3.23411	2.78344	0.25103	2.51033
Ex 10days	0.351	6.31534	6.71126	4.35997	1.52033	2.47996	0.86004	2.47356	5.41520	2.50600	0.31775	4.22732	2.86804	0.24369	3.42836
Ex 10days	0.175	5.89068	5.38702	4.92146	1.22380	1.87429	0.91476	2.83485	6.09590	2.47596	0.26117	3.74970	2.76415	0.26117	2.89293
Ex 10days	0.257	5.76926	6.10154	5.91427	1.27761	2.16871	0.88235	2.90382	5.40740	2.76708	0.30020	4.09271	2.82594	0.35810	3.20878
Ex 10days	0.241	7.44391	4.48522	7.20660	1.15448	2.18461	0.89752	2.87226	6.29070	3.08817	0.29141	4.00096	3.04626	0.41630	3.34809
Ex 10days	0.390	7.87329	3.36076	5.34783	1.30509	1.94957	0.81479	2.48466	6.82180	2.92113	0.51538	4.02826	2.93244	0.46583	3.12104
Ex 10days	0.191	6.73652	5.67848	6.82157	1.82890	2.51184	1.08313	2.91886	6.43960	3.44140	0.24978	4.79776	3.84702	0.34328	3.98697
Ex 10days	0.232	6.57792	3.59262	5.95670	1.61788	2.25386	0.58790	2.54614	7.93550	2.83608	0.32958	3.14604	2.82967	0.25280	2.57696
Ex 10days	0.178	6.78738	4.82226	5.47212	1.32244	2.03494	0.73166	2.20923	6.15140	3.53554	0.35052	4.51425	3.43238	0.39370	3.34296

Ex 10days	0.201	6.30088	4.06063	6.67067	1.55708	2.00874	0.72855	2.16328	6.65270	3.12024	0.30678	4.52016	3.04420	0.42183	3.36596
Ex 10days	0.215	5.50444	5.54076	5.11142	1.43371	2.03917	0.85762	2.53351	7.14860	2.91412	0.39093	3.78856	3.08064	0.39093	3.37230
Ex 10days	0.259	5.66862	5.41658	4.73669	1.29141	1.95837	0.76588	2.34804	6.99210	3.39561	0.43437	3.92053	3.65447	0.40049	3.32755
Ex 10days	0.207	5.85388	5.76152	6.01194	1.48864	2.00425	0.94952	3.17016	7.66160	2.65660	0.29463	3.14021	2.88769	0.26354	2.40372
Ex 10days	0.176	5.25565	5.36305	5.77887	1.86704	1.72291	0.76583	2.40733	6.01300	2.61565	0.22148	3.54619	2.84719	0.19425	2.73319
Ex 10days	0.260	6.62349	6.90371	6.06018	1.51299	2.39217	1.03465	3.14025	6.50020	2.69704	0.30307	2.94244	2.85009	0.38383	2.18822
Ex 10days	0.251	6.08280	7.73730	6.13676	1.43523	2.51724	0.84982	3.24485	5.55010	2.95400	0.33113	4.23100	2.85761	0.35852	3.61467
Ex 10days	0.197	7.43115	2.60185	6.47265	1.72000	1.97545	0.64487	2.48466	5.98090	3.32261	0.34618	4.54261	3.20357	0.44768	3.76961
Ex 10days	0.182	7.08592	3.47863	5.98122	1.11008	1.63608	0.82601	3.04021	6.06850	2.71875	0.32958	3.73931	2.93102	0.44684	3.22493
Ex 10days	0.254	6.22496	4.56878	5.33066	1.53884	1.83946	0.83180	2.09670	6.56410	3.66725	0.36880	4.95837	3.74697	0.32984	4.41360
Ex 10days	0.165	6.03683	4.73946	5.50156	1.56079	2.39232	0.86686	2.68296	7.56670	3.08767	0.43531	3.59202	3.19687	0.60640	3.19687
Ex 10days	0.220	7.26867	3.44917	5.75550	1.37438	1.86778	0.83104	2.68584	6.89630	3.20385	0.24000	3.63354	3.10362	0.36880	2.91333
Ex 10days	0.147	5.94126	6.09694	5.99321	1.38677	2.28307	0.82679	2.36090	6.64070	3.01911	0.32907	3.78749	3.12110	0.39903	2.99628
Ex 10days	0.175	6.90725	4.71404	5.80162	1.69188	1.80915	0.85941	2.34496	7.34630	2.71346	0.37500	3.75371	2.63325	0.25000	3.17350
Ex 10days	0.186	7.16044	3.64964	5.44752	1.43704	1.76875	0.74435	2.51805	6.88660	2.54387	0.46021	4.08859	2.66190	0.41875	3.25120
Ex 10days	0.212	6.90321	4.59017	6.29112	1.30263	2.26279	0.74060	2.75268	6.28730	2.88444	0.34513	4.48644	3.04104	0.34513	3.68020
Ex 10days	0.326	5.60667	5.84863	6.07790	1.37442	2.19460	0.65725	2.81453	6.92810	3.46191	0.49948	4.06400	3.52844	0.45407	3.38348
Ex 10days	0.252	5.83931	5.29949	5.33320	1.20465	2.21770	0.73514	2.32642	7.25090	2.79289	0.43352	4.07112	2.92915	0.37770	3.13219
Ex 10days	0.251	6.18326	3.91257	5.51216	0.97271	1.58478	0.75466	2.68140	6.61590	2.92361	0.47467	4.29175	2.89622	0.63411	3.22601
Ex 10days	0.342	6.51352	5.12198	5.21850	1.21442	1.80813	0.90026	2.88220	6.63260	3.52382	0.15270	4.82118	3.69191	0.23426	3.65932
Ex 10days	0.160	4.10200	6.51210	4.92259	1.25440	1.74563	0.74591	2.44429	7.47080	2.93358	0.24978	3.74258	2.84375	0.30307	2.93358
Ex 10days	0.232	5.08314	5.49756	5.33421	0.76604	2.07285	0.79756	2.38902	6.38730	3.17073	0.37729	3.67195	3.33979	0.29463	3.17730
Ex 10days	0.259	5.93379	4.82254	4.75163	1.88350	1.83940	0.68786	1.92925	7.21160	2.66241	0.37500	3.59504	2.81670	0.38417	2.77791
Ex 10days	0.232	8.38330	1.92530	5.23061	0.76940	1.98722	0.40460	2.13064	6.05660	2.56382	0.12638	3.79888	2.79719	0.16479	3.23502
Ex 10days	0.315	7.35456	4.55652	7.37803	1.15080	2.00723	0.69760	2.81985	6.53050	3.06442	0.32145	3.75117	3.12304	0.32145	2.98200
Ex 10days	0.168	6.70939	4.74341	5.84358	1.46251	1.92000	0.82759	2.79823	7.00300	3.38112	0.29141	4.41688	3.45972	0.29437	3.71848
Ex 5day-Out	0.231	7.14900	3.94215	7.18309	1.75265	2.16436	0.73467	2.43201	6.56950	2.77404	0.31622	4.52477	2.84615	0.57953	3.07715
Ex 5day-Out	0.201	8.28310	3.19487	6.57992	1.75316	2.13834	0.69856	2.21897	7.04990	3.13859	0.37948	4.66296	3.21606	0.45608	3.50980
Ex 5day-Out	0.266	7.03831	5.44939	6.18454	1.61467	2.50051	1.00284	2.74515	7.69280	2.95763	0.32000	5.11655	2.87770	0.52000	3.83881
Ex 5day-Out	0.230	8.02135	4.11209	6.29948	1.64188	2.44329	0.72934	2.40340	7.14050	2.82842	0.20000	4.40715	3.12865	0.31240	3.45404
Ex 5day-Out	0.321	7.15108	5.68422	5.89115	1.46752	2.20036	0.66662	3.02255	6.50970	3.16708	0.26832	4.56156	3.20524	0.35776	3.36476
Ex 5day-Out	0.242	7.73050	4.74527	6.29167	1.43176	2.30335	0.72404	2.67541	6.51020	3.30149	0.32107	4.82169	3.39249	0.55242	3.47737
Ex 5day-Out	0.266	7.21375	3.86682	6.12558	1.25071	1.89470	0.78946	2.32922	6.39540	3.06330	0.33333	4.11483	3.23174	0.50000	2.70439
Ex 5day-Out	0.232	6.09333	4.96254	6.64585	1.69516	1.94685	0.86624	3.01708	6.91740	3.24287	0.32862	5.29803	3.20311	0.45019	4.02760

Ex 5day-Out	0.235	6.52479	4.57250	7.03108	0.98885	1.80846	0.73480	2.69552	6.91330	3.41096	0.37500	3.94204	3.21759	0.29463	3.28938
Ex 5day-Out	0.270	6.88462	6.50518	6.19743	1.25000	2.36499	0.97548	3.23391	7.86530	3.19048	0.33219	4.23208	3.53136	0.37599	3.60376
Ex 5day-Out	0.199	6.28915	5.44325	6.48141	1.21739	1.90277	0.84194	1.91047	7.30240	3.63029	0.38383	4.58729	3.63438	0.41838	3.63438
Ex 5day-Out	0.235	7.34011	4.44035	6.56255	1.16620	2.34911	0.79016	2.80776	7.70600	3.16984	0.42969	3.97856	3.04472	0.53807	3.20924
Ex 5day-Out	0.297	6.46094	5.13116	5.65179	1.77712	2.26575	0.78617	2.27683	5.62850	3.14836	0.21718	4.71978	3.02414	0.35818	3.22754
Ex 5day-Out	0.235	6.34805	3.49575	6.10957	1.07031	1.94018	0.76246	2.64652	5.35260	3.25938	0.39495	4.42343	3.68424	0.37696	3.27048
Ex 5day-Out	0.199	6.87899	4.52739	5.95713	1.40868	1.84039	0.69333	2.39092	6.49290	3.88939	0.44720	5.81209	4.10582	0.37948	4.37433
Ex 5day-Out	0.246	7.54273	1.75987	7.27212	1.55221	1.81902	0.60105	2.24365	7.22900	3.07768	0.21246	3.42040	3.05600	0.29463	2.63212
Ex 5day-Out	0.195	6.61407	3.62918	6.66200	1.80666	1.70699	0.79883	2.41820	7.52460	3.63209	0.49658	4.28191	3.69973	0.58833	3.33400
Ex 5day-Out	0.222	6.09237	5.25143	6.45896	0.89582	2.20363	0.54804	2.05567	6.56510	3.03062	0.54478	4.67123	2.99557	0.52535	2.93641
Ex 5day-Out	0.221	5.99332	4.71168	5.26081	0.93027	1.85600	0.73475	2.39557	7.19760	3.23189	0.42308	4.63706	3.23189	0.42308	3.40826
Ex 5day-Out	0.216	7.51504	3.48658	5.45069	1.21333	1.94980	0.60382	2.10430	7.47610	3.84850	0.32062	3.73096	2.84719	0.35802	3.19254
Ex 5day-Out	0.250	5.52884	7.19026	6.19075	1.38508	2.36010	0.93624	2.42935	7.32120	2.42563	0.27176	3.59879	2.56342	0.31692	2.91129
Ex 5day-Out	0.243	5.26133	6.17847	6.52033	0.61974	2.18733	1.03066	2.70167	6.96010	2.81939	0.35600	3.83622	2.96991	0.34358	2.54448
Ex 5day-Out	0.202	6.84781	6.32049	6.04854	1.28831	2.12048	0.91411	3.03455	5.35160	3.42160	0.26354	4.12306	3.43671	0.22438	3.03592
Ex 5day-Out	0.225	5.82579	5.88141	5.43015	1.14971	2.32194	0.77445	2.95363	6.42130	2.76923	0.27196	3.19600	2.88565	0.38462	2.57950
Ex 5day-Out	0.204	6.26164	6.41966	6.25646	1.08725	2.07761	0.81925	2.60419	7.20430	2.81669	0.27474	4.43713	2.87182	0.31622	3.24715
Ex 5day-Out	0.135	6.54057	4.64423	5.38188	1.33592	2.14220	0.64914	2.45686	6.59850	3.33275	0.25324	4.39707	3.30877	0.26331	3.47134
Ex 5day-Out	0.188	7.19074	3.46416	4.81537	1.55447	1.50385	0.83225	2.38809	6.34970	2.56280	0.50146	3.48576	2.48804	0.53846	2.65088
Ex 48hr	0.342	7.16602	5.52678	6.34171	1.50087	2.49793	0.73915	2.41344	6.95640	2.94188	0.43462	4.90129	2.93092	0.46543	3.44021
Ex 48hr	0.250	7.65439	4.33211	5.85389	1.74617	2.19034	0.61363	2.33953	6.63580	3.14352	0.53727	4.23964	3.16304	0.49527	3.05940
Ex 48hr	0.220	7.07217	4.57560	6.47896	1.58686	2.25284	0.65968	2.58240	6.55090	3.48858	0.33562	4.54913	3.61254	0.33562	3.31792
Ex 48hr	0.271	7.42376	4.19342	6.72709	1.56052	1.99919	0.72311	2.46709	7.01170	3.08416	0.33304	4.56440	3.24224	0.45981	3.64352
Ex 48hr	0.264	7.39452	6.01318	6.04567	1.37908	2.33852	0.65552	2.69189	7.42420	2.93504	0.32907	4.51200	3.05100	0.39903	3.29048
Ex 48hr	0.239	9.03744	4.16992	6.74328	1.45892	2.31969	0.85232	3.04297	7.41610	3.40639	0.20815	3.90362	3.05400	0.37696	3.21539
Ex 48hr	0.299	7.79179	3.67046	6.30570	1.96087	1.87683	0.62500	2.50033	7.45780	2.88000	0.40000	4.68016	3.12024	0.40000	3.16100
Ex 48hr	0.248	7.54835	4.43948	6.36700	1.94054	2.58641	0.87370	2.49908	7.26930	2.79572	0.30744	4.54120	3.11540	0.27500	3.39600
Ex 48hr	0.319	7.99328	3.48702	6.56048	1.77413	2.21933	0.71163	2.37663	5.44260	2.92408	0.37500	4.70239	2.81008	0.41667	3.15762
Ex 48hr	0.271	7.37237	6.90793	5.91516	1.60339	2.79488	0.90653	3.16106	6.04360	3.01963	0.17888	3.78342	3.26225	0.21540	2.72367
Ex 48hr	0.234	7.50432	4.55600	6.33389	1.66989	2.31649	0.65816	2.92114	6.40990	3.44462	0.23077	3.95481	3.42135	0.23077	3.25835
Ex 48hr	0.201	7.96252	4.44020	7.27694	1.19231	2.38376	0.72237	2.34276	7.28170	3.52000	0.24332	5.12064	3.52024	0.36000	3.72000
Ex 48hr	0.185	8.16906	3.01824	6.80149	1.51715	1.99962	0.74985	2.62795	7.13930	2.92928	0.35052	4.04558	3.40240	0.56687	3.13110
Ex 48hr	0.176	8.94830	1.27890	6.80072	2.00421	1.81950	0.80504	2.26050	8.50260	3.44199	0.36489	5.14820	3.64947	0.37881	3.77736
Ex 48hr	0.218	7.30152	5.92698	6.43404	1.63988	2.36629	0.83249	2.50612	6.60810	2.83521	0.20712	4.50524	3.11751	0.31715	3.43749

Ex 48hr	0.209	5.64017	5.78133	6.40554	1.57692	2.42585	0.67279	2.51631	6.28690	1.77960	0.84367	2.38773	2.96110	0.33333	3.26559
Ex 48hr	0.204	8.86468	3.81364	6.12714	1.50109	1.94565	0.73961	2.21471	6.11200	1.88508	0.82581	2.29120	3.31904	0.37881	3.48392
Ex 48hr	0.289	7.59485	6.39415	6.60015	1.25220	2.26612	0.74072	2.68328	7.42950	1.76408	0.84000	2.51076	3.22116	0.52312	3.92020
Ex 48hr	0.291	6.52792	5.80758	6.43054	1.86569	2.29064	0.76933	2.72592	8.10240	2.00949	0.67431	2.63719	3.18630	0.37364	4.17489
Ex 48hr	0.138	6.99834	4.77756	6.31567	0.93296	2.15948	0.72784	2.25963	5.61180	1.89817	0.66998	2.18822	3.24722	0.43046	2.91661
Ex 48hr	0.221	6.86059	3.74441	5.92869	1.82950	2.09548	0.66900	1.99348	6.85070	1.90958	0.80076	1.95854	2.76415	0.29437	2.96454
Ex 48hr	0.302	6.88093	5.24567	5.57891	0.85809	2.10411	0.77875	2.92152	6.33760	1.82763	0.58900	2.40404	3.45708	0.36421	3.84779
Ex 48hr	0.254	6.12542	4.26489	5.97648	1.34950	2.04000	0.72327	2.37506	7.04130	1.70712	0.68997	1.01615	2.90708	0.32222	3.00419
Ex 48hr	0.292	6.25931	6.23949	5.17635	1.56100	2.18429	0.70944	3.12978	5.94320	1.47512	0.82722	2.10524	2.86356	0.35852	3.35808
Ex 48hr	0.191	5.93281	5.55539	4.66500	1.26614	2.12748	0.63453	2.09282	6.73750	1.58388	0.42969	2.08375	2.94188	0.25781	3.27450
Ex 48hr	0.245	6.98445	5.35665	5.60464	1.14544	2.19579	0.92682	2.65215	6.51740	2.25500	0.60879	2.36658	3.11192	0.34149	2.88825
Ex 48hr	0.348	6.96780	5.19450	5.46772	0.97246	2.23871	0.62847	2.50094	7.02580	1.89817	0.61056	2.45487	3.25422	0.35776	2.63213
Ex 48hr	0.221	6.87638	5.33352	5.81621	2.07636	2.03749	0.92979	2.92449	6.46770	1.90804	0.71138	1.79217	3.02333	0.30307	2.85833
Ex 24hr	0.279	7.00635	5.97795	6.50908	1.62071	2.80717	0.42584	2.67067	7.27160	3.34608	0.41727	4.89350	3.35438	0.41727	3.84079
Ex 24hr	0.305	7.16635	4.44225	6.05004	1.29771	1.87650	0.78650	2.59744	7.37530	2.64273	0.40168	3.96671	2.68261	0.40168	3.08685
Ex 24hr	0.218	7.83433	4.25000	6.16052	1.59872	2.00766	0.80698	2.32077	6.42240	2.96096	0.50175	4.62800	3.00000	0.54325	3.16667
Ex 24hr	0.251	8.33998	2.81312	7.07072	1.40572	1.97677	0.79643	2.39990	7.43240	3.13804	0.37729	4.99392	3.17325	0.51538	3.38554
Ex 24hr	0.212	7.11194	3.03166	5.81652	1.99280	2.01815	0.83593	2.54849	6.92100	3.09848	0.34375	4.25787	3.05044	0.42969	3.05907
Ex 24hr	0.220	7.76837	3.58143	7.58176	1.36236	1.84154	0.79241	2.38847	7.42780	3.33979	0.32145	5.06199	3.26019	0.36105	3.80296
Ex 24hr	0.246	7.32474	3.74863	5.67751	1.51363	2.02257	0.52078	2.35090	7.61540	3.09896	0.20712	4.27389	3.21541	0.23396	3.36304
Ex 24hr	0.216	7.58762	4.19508	6.13240	1.40000	2.33840	0.77947	2.63251	7.24560	3.00023	0.36000	4.38531	2.88462	0.32000	3.76942
Ex 24hr	0.165	6.91026	4.54118	5.85267	1.34500	2.47922	0.75361	3.02845	7.62770	3.00146	0.26354	3.95129	3.08250	0.34358	3.17325
Ex 24hr	0.188	8.26817	3.59229	5.92913	1.14313	1.93023	0.72106	2.24553	6.29700	2.95900	0.13869	3.08961	2.82481	0.19231	2.34565
Ex 24hr	0.316	7.08712	4.13416	6.15251	1.27933	2.24406	0.64299	2.61111	5.82170	3.09608	0.17888	3.52612	3.18673	0.37736	2.88258
Ex 24hr	0.280	6.39896	4.25554	7.03678	1.38741	1.97802	0.65933	2.35794	6.59610	2.99767	0.37696	4.52413	3.12781	0.33562	3.31477
Ex 24hr	0.252	8.07064	2.49226	5.96400	1.44879	1.80525	0.70937	1.45922	7.39020	2.94612	0.28000	3.32065	2.88047	0.32862	2.77201
Ex 24hr	0.202	6.87824	4.62956	5.01949	0.98585	2.40750	0.72570	2.01189	6.68920	2.67577	0.31692	3.55181	2.43254	0.27980	2.63931
Ex 24hr	0.209	7.15029	4.69101	4.80927	1.09054	1.92210	0.77650	2.29460	7.44610	2.90132	0.26354	3.19248	2.61012	0.30333	2.45600
Ex 24hr	0.239	7.22496	3.20871	6.31508	1.70153	1.95369	0.62865	2.43795	7.98640	3.08773	0.24332	3.71308	3.22892	0.24332	3.07042
Ex 24hr	0.201	7.34837	3.67882	6.22746	1.09462	1.96164	0.63244	2.42156	8.00940	3.06969	0.25324	4.17392	2.92158	0.33562	3.28615
Ex 24hr	0.269	7.08578	4.28872	5.35536	0.88546	2.50158	0.79377	2.16503	######	3.05695	0.27196	3.41767	3.01779	0.31008	3.20668
Ex 24hr	0.228	7.07413	3.78642	5.74736	1.07982	1.98312	0.67554	2.34483	7.63360	2.81824	0.36851	3.06620	2.69072	0.36851	2.20908
Ex 24hr	0.270	7.50847	5.14973	5.81096	1.46611	2.56412	0.51276	2.01314	8.38030	2.43558	0.20000	3.04308	2.55039	0.32000	2.41419
Ex 24hr	0.273	6.70296	5.53364	6.18467	1.09836	1.88664	0.84753	3.00934	7.58560	3.15183	0.29120	4.04155	3.21094	0.25300	3.65518

Ex 24hr	0.208	7.16300	3.00108	5.28396	1.47783	2.11771	0.87922	2.62910	7.36130	2.68114	0.25346	3.12926	2.77050	0.34358	2.55608
Ex 24hr	0.156	8.04144	1.22336	5.85024	1.44056	1.83911	0.71898	1.75930	7.91860	3.14271	0.34176	3.77308	3.05988	0.30464	3.01413
Ex 24hr	0.118	7.51663	3.98118	5.57887	1.48844	1.96768	0.69678	2.04943	6.52170	2.72838	0.39093	3.98581	2.90532	0.34749	2.80632
Ex 24hr	0.202	7.41760	4.13784	6.61226	1.11572	1.93999	0.65995	2.29110	8.60780	3.08318	0.15987	4.28975	3.16419	0.32222	3.28904
Ex 24hr	0.162	7.53289	2.09761	6.47358	0.62096	1.91419	0.57177	1.96454	6.55390	3.18333	0.16667	4.16750	3.32629	0.29167	3.15458
Ex 24hr	0.251	6.04012	3.84188	4.38461	1.28000	2.05983	0.62381	1.92733	6.15450	3.03601	0.21725	3.14732	3.18593	0.25254	2.76099
Ex 24hr	0.188	6.31459	4.71001	5.00176	1.08413	1.74095	0.59813	2.44281	5.64150	2.88002	0.35083	3.04925	3.01149	0.20411	2.30815
Ex 24hr	0.197	6.93304	3.22116	4.53768	1.12924	1.96438	0.58580	2.41399	4.91550	2.56557	0.22222	3.26117	2.69600	0.29630	2.56670
Ex 24hr	0.367	7.23904	3.06969	5.46263	0.97452	1.72378	0.79812	3.28816	6.77050	2.79205	0.27196	3.45879	2.91439	0.30769	2.59333
Ex 24hr	0.275	6.09785	4.87615	5.60908	1.70880	2.33198	0.61936	2.01922	5.23160	2.61875	0.20696	2.52782	2.65719	0.29271	1.99840
Ex 24hr	0.225	6.78812	4.40558	5.68970	0.91227	2.31212	0.59427	2.47934	5.87380	2.75536	0.21540	3.47908	2.63696	0.24000	2.26876
Ex 24hr	0.247	7.78531	4.24823	5.98376	1.32968	2.11072	0.78727	2.65112	4.92990	2.76819	0.28284	4.19684	3.18593	0.28284	3.48985
Ex 24hr	0.313	7.51103	3.81731	5.61391	1.31940	2.45740	0.73951	2.62449	6.59880	2.73014	0.29097	3.71723	2.57850	0.34149	2.84448
Ex 24hr	0.226	7.34020	3.76128	5.43817	1.34460	2.04728	0.64236	2.08088	7.81310	3.18044	0.42873	4.68392	3.33948	0.40035	3.79052
Ex 24hr	0.248	7.58761	3.50923	5.56864	1.15246	2.08011	0.54465	1.53620	6.99750	2.46740	0.19231	3.54400	2.82420	0.27196	2.87360
Ex 24hr	0.184	7.77392	4.29833	5.50963	1.38689	2.47481	1.87724	2.27292	6.70020	3.00029	0.27196	3.38629	3.15842	0.28000	3.19204
Ex 24hr	0.214	7.40788	6.14702	6.72526	1.46154	2.40816	0.77551	3.06151	6.56700	2.74636	0.31240	3.74488	2.98932	0.28844	3.25968
Ex 24hr	0.197	6.87385	3.04989	5.01492	1.29004	2.02283	0.48024	1.49791	6.22120	2.61565	0.36851	3.03554	2.72835	0.40759	2.14285
Ex 24hr	0.163	6.48080	4.95600	4.19854	0.85796	2.03198	0.84845	2.57286	6.94420	2.92857	0.28000	3.41814	2.84125	0.32862	2.69661
Ex 24hr	0.211	6.93600	4.23708	4.88788	1.23225	2.09790	0.78386	2.90027	6.35690	2.60814	0.31008	3.36581	2.53262	0.31008	2.25340
Ex 24hr	0.153	6.83123	6.05217	5.57479	0.86431	2.28408	0.85900	2.88013	7.40890	2.87136	0.23413	3.62084	2.78280	0.29165	2.39100
Ex 24hr	0.181	7.54440	3.66346	5.73285	0.92000	2.12134	0.75993	2.80432	7.25380	3.00000	0.27474	3.72088	2.96028	0.27474	3.04000
Ex 24hr	0.177	6.18530	5.65520	5.31008	1.45892	1.97339	0.72477	2.61847	6.92300	2.86216	0.30769	3.61508	2.84452	0.34615	2.70172
