# Supplementary material for: Novel predator-induced phenotypic plasticity by hemoglobin and physiological changes in the brain of Xenopus tropicalis
Source: Front Physiol. 2023 Jun 6;14:1178869. doi: 10.3389/fphys.2023.1178869 (PMC10279953; doi:10.3389/fphys.2023.1178869)
Supplement: Supplementary file 4 [file Table2.pdf]

**Supplementary Table 2.** Primers used for real-time PCR.

| Primer | |
|---|---|
| *HBA3* Forward | 5'-GAATGCATCAGCAAGGGCAG-3' |
| *HBA3* Reverse | 5'-CAAGTCAACACAAGTGCAACAACG-3' |
| *ALAS2* Forward | 5'-GCTACCCTCAAGCAACAGTG-3' |
| *ALAS2* Reverse | 5'-GCAACACAGCACTGATGTG-3' |
| *PYGM* Forward | 5'-GATTCCATACCCATAAGCCG-3' |
| *PYGM* Reverse | 5'-GATTGACCAGATCAGGGATG-3' |
| *G6PC3* Forward | 5'-CATAAAATTCAGTCCACAGGATGGC-3' |
| *G6PC3* Reverse | 5'-CTAACTTAGTCCTGACTCCTCGTTC-3' |
| *FAH* Forward | 5'-GAGGACATTCCTGCAAGATG-3' |
| *FAH* Reverse | 5'-GTCTCTGCAACCTCTGAGTG-3' |
| *PDK4* Forward | 5'-CCTGTCTTATGCTGTTGTAGTGG-3' |
| *PDK4* Reverse | 5'-GCTTGACGTGGAGACACAATG-3' |
| *SOD3* Forward | 5'-CACACTGACTTGCGCTCTCTG-3' |
| *SOD3* Reverse | 5'-CGACTGGCTATGCTGAACAG-3' |
| *PSAT* Forward | 5'-CCTGTTTGCTAAAGGGACC-3' |
| *PSAT* Reverse | 5'-GCAGGTACCAACCAGTTGGTTC-3' |
| *TYR* Forward | 5'-GCCTTGAGAGGAAAGTCATATTGC-3' |
| *TYR* Reverse | 5'-GTACATATAGGGATGGCTGGGC-3' |
| *SDF4* Forward | 5'-CTGCAGTACTTTGAATAGGCAGC-3' |
| *SDF4* Reverse | 5'-CTGATCTTTTCAACCTGCTCTCAG-3' |
