# Supplementary material for: Novel predator-induced phenotypic plasticity by hemoglobin and physiological changes in the brain of Xenopus tropicalis
Source: Front Physiol. 2023 Jun 6;14:1178869. doi: 10.3389/fphys.2023.1178869 (PMC10279953; doi:10.3389/fphys.2023.1178869)
Supplement: Supplementary file 5 [file Table6.pdf]

Supplementary Table. 6
Diseases and Bio Functions predicted by IPA. All altered diseases and bio functions used in Fig.7b.
© 2000-2019 QIAGEN. All rights reserved.

Diseases and Bio Functions	6hr/Cont	24hr/Cont	48hr/Cont	10day/Cont	5day-O/Cont	5days-O/10days
Organismal death	3.745	5.768	5.969	11.299	8.921	1.173
Morbidity or mortality	N/A	5.776	5.969	11.336	N/A	1.249
Cell proliferation of tumor cell lines	-3.515	-5.622	-4.885	-6.052	-4.092	N/A
Cell viability of tumor cell lines	-4.216	-4.454	-3.69	-6.317	-3.893	N/A
Cell survival	-3.532	-4.117	-3.082	-6.439	-4.804	N/A
Cell viability	-3.516	-4.03	-3.099	-6.399	-4.491	N/A
Necrosis	2.031	4.602	3.474	4.881	3.479	1.374
Apoptosis	2.144	3.887	2.49	5.075	3.104	0.875
Formation of gamma H2AX nuclear focus	2.563	2.823	2.758	3.638	3.513	N/A
Cell death of tumor cell lines	2.107	3.157	3.071	4.1	2.364	N/A
Repair of DNA	-2.493	-2.906	-2.549	-3.453	-1.485	N/A
Homologous recombination	-2.362	-3.052	-3.228	-2.773	-1.394	N/A
Cycling of centrosome	-2.06	-3.009	-2.675	-2.688	-1.852	N/A
Incidence of tumor	2.348	2.946	2.646	0.8	1.235	2.066
Cytokinesis	-2.98	-2.486	-2.35	-2.394	-1.263	0.41
Entry into interphase	-1.973	-2.77	-2.989	-2.883	-1.23	N/A
Digestive system cancer	-2.621	-2.038	-1.689	-3.166	-1.866	N/A
Cell death of cervical cancer cell lines	2.441	2.402	1.854	3.103	1.421	N/A
Chromosomal aberration	2.27	2.762	1.932	2.407	1.804	N/A
Frequency of tumor	2.349	2.662	2.177	0.815	0.845	2.064
Cell cycle progression	-0.898	-2.179	-1.901	-3.649	-2.262	N/A
Recombination of cells	-1.97	-3.058	-2.547	-3.211	N/A	N/A
Development of malignant tumor	2.138	2.793	2.284	1.038	1.63	0.9
Apoptosis of tumor cell lines	1.406	1.855	1.959	3.099	1.995	0.348
Growth of organism	N/A	-2.134	N/A	-4.156	-4.288	N/A
Growth of connective tissue	-2.106	N/A	-2.012	-3.344	-3.079	N/A
Metabolism of DNA	-2.754	-2.658	-1.814	-2.162	-0.677	-0.38
Hepatobiliary system cancer	-2.213	-2.194	-1.706	-3.28	-1.002	N/A
Tumorigenesis of reproductive tract	2.433	2.138	2.138	1.772	1.89	N/A

Development of lung tumor	1.827	2.769	2.284	1.722	1.668	N/A	
Association of chromosome components	N/A	-2.496	-2.789	-2.705	-2.208	N/A	
Cytokinesis of tumor cell lines	-2.961	-1.761	-1.864	-1.674	-1.914	N/A	
DNA replication	-2.081	-2.516	-2.003	-2.646	-0.797	N/A	
Apoptosis of cervical cancer cell lines	2.81	N/A	2.194	3.134	1.811	N/A	
Interphase	-2.256	-2.505	-2.165	-1.985	-0.951	N/A	
Aplasia or hypoplasia	N/A	N/A	N/A	5.376	4.34	N/A	
Hypoplasia	N/A	N/A	N/A	5.291	4.34	N/A	
Abdominal cancer	-1.744	-1.783	-0.911	-2.8	-1.207		1.109
Adenocarcinoma	1.551	2.356	2.24	1.867	1.488	N/A	
Formation of mitotic spindle	1.715	1.826	1.854	2.159	1.92	N/A	
Development of adenocarcinoma	1.644	2.107	2.188	1.963	1.562	N/A	
Alignment of chromosomes	-2.345	-2.538	-2.137	-2.331	N/A	N/A	
Liver tumor	-1.354	-1.819	-1.457	-2.528	-0.199		1.951
Female genital neoplasm	2.219	1.912	1.912	1.554	1.633	N/A	
G1/S phase transition	-1.938	-1.852	-1.852	-1.957	-1.507	N/A	
M phase	-2.518	-1.837	-1.883	-1.767	-0.963		0.078
Senescence of cells	N/A	2.25	2.679	2.58	1.441	N/A	
DNA recombination	-2.19	-1.837	-1.634	-1.706	-1.564	N/A	
M phase of tumor cell lines	-2.724	-1.632	-2.013	-1.313	-1.108	N/A	
Formation of nuclear foci	1.221	1.899	1.613	2.127	1.845	N/A	
Respiratory system tumor	1.328	1.783	2.289	0.466	0.399		2.415
Digestive organ tumor	-1.114	-1.017	-1.065	-2.041	-0.868		2.42
Hepatocellular carcinoma	-1.982	-1.754	-1.142	-3.231	-0.387	N/A	
Formation of spindle apparatus	1.452	1.566	1.611	1.982	1.839	N/A	
Proliferation of connective tissue cells	N/A	N/A	-2.089	-3.309	-3.048	N/A	
Missegregation of chromosomes	1.755	1.834	1.834	1.486	1.517	N/A	
Lung adenocarcinoma	1.219	1.718	1.551	1.984	1.915	N/A	
DNA damage	-0.629	1.897	1.233	2.649	1.957	N/A	
Chromosomal congression of chromosomes	-2.433	-1.912	-1.912	-1.912	N/A	N/A	
Cell proliferation of fibroblasts	-2.444	N/A	N/A	-2.802	-2.868	N/A	
Hepatobiliary carcinoma	-1.982	-1.489	-0.829	-3.099	-0.434	N/A	
Liver cancer	N/A	-2.194	-1.706	-3.129	-0.743	N/A	
Non-small cell lung carcinoma	1.23	1.723	1.559	1.693	1.534	N/A	

Organization of cytoskeleton	−2.083	N/A	0.647	−3.238	−1.758	N/A
Polyploidy	N/A	2.394	2.175	2.594	0.536	N/A
Checkpoint control	−2.2	−1.579	−1.04	−1.542	−1.29	N/A
Mitotic index of cervical cancer cell lines	N/A	−1.845	−1.845	−1.845	−1.998	N/A
Association of chromatin	N/A	−2.771	N/A	−2.952	−1.793	N/A
S phase	N/A	−1.76	−2.213	−1.89	−1.559	N/A
Liver carcinoma	−1.982	−1.489	−0.829	−2.936	−0.136	N/A
Microtubule dynamics	−2.434	N/A	N/A	−3.369	−1.561	N/A
Cell proliferation of breast cancer cell lines	−1.778	N/A	−3.278	−2.276	N/A	N/A
Segregation of chromosomes	−0.745	−1.321	−1.789	−1.689	−0.792	0.928
Organization of cytoplasm	−2.083	N/A	N/A	−3.238	−1.758	N/A
Chromosomal instability	1.86	2.138	N/A	1.77	1.268	N/A
Tumorigenesis of epithelial neoplasm	1.439	1.195	1.181	−0.689	0.833	1.667
Aneuploidy	0.739	1.942	1.497	2.477	0.317	N/A
Gonadal tumor	2.219	N/A	1.937	2.164	0.64	N/A
Lung carcinoma	1.309	1.775	1.621	0.74	1.454	N/A
Malignant solid tumor	0.858	1.046	1.231	−0.391	−0.85	2.464
Atrophy of testis	1.557	N/A	2.262	N/A	2.917	N/A
Lung cancer	1.145	1.276	1.791	−0.011	0.231	2.207
Ovarian tumor	2.219	N/A	1.664	1.706	1	N/A
Thoracic neoplasm	1.328	1.863	2.289	0.647	0.37	N/A
Hematologic cancer of cells	N/A	2.048	1.762	1.004	1.679	N/A
Genital tumor	1.455	1.294	1.854	0.913	0.921	N/A
Lung tumor	1.328	1.783	2.289	0.606	0.399	N/A
Benign solid tumor	N/A	1.139	−0.274	−1.331	0.854	2.774
Lymphocytic cancer	N/A	1.99	1.698	0.973	1.679	N/A
Lymphocytic neoplasm	N/A	1.99	1.698	0.808	1.842	N/A
Lymphoproliferative disorder	N/A	1.99	1.698	0.808	1.842	N/A
M phase of cervical cancer cell lines	−2.359	−0.941	−1.332	−0.886	−0.816	N/A
Neoplasia of leukocytes	N/A	1.955	1.66	0.79	1.842	N/A
Extracranial solid tumor	0.512	0.156	0.446	−0.744	−1.195	3.105
Spindle checkpoint of cells	−1.192	−1.309	−1.192	−0.655	−1.698	N/A
Mitosis	−0.405	−1.007	−1.133	−1.735	−1.743	N/A
Recombination	N/A	−1.754	−1.634	−1.35	−1.247	N/A

Attachment of kinetochores	N/A	−1.982	−1.982	−1.982	N/A	N/A
Breast or pancreatic cancer	1.206	1.342	1.026	0.897	−1.373	N/A
Muscle contraction	N/A	2.433	N/A	1.342	N/A	−1.982
Synthesis of DNA	N/A	N/A	−1.144	−3.062	−1.498	N/A
Head and neck tumor	1.982	0.643	−0.83	0.374	0.714	1.154
Contractility of muscle	2.905	N/A	N/A	N/A	N/A	−2.762
Proliferation of fibroblast cell lines	N/A	N/A	N/A	−3.295	−2.341	N/A
Abdominal carcinoma	−1.982	−0.691	0.064	−2.742	0.086	N/A
Development of carcinoma	1.562	1.308	1.572	0.004	1.074	−0.025
Lymphoid cancer	N/A	1.737	1.418	0.491	1.842	N/A
Secondary tumor	N/A	N/A	−0.789	−2.755	−0.498	1.419
Hematologic cancer	N/A	2.318	N/A	1.276	1.825	N/A
Hematopoietic neoplasm	N/A	2.354	N/A	1.042	2.019	N/A
Dysgenesis	N/A	N/A	N/A	5.376	N/A	N/A
Mammary tumor	0.558	−0.424	−0.46	−0.662	−2.237	1.004
Pelvic tumor	1.664	1.066	1.394	0.291	0.913	N/A
Non−melanoma solid tumor	0.816	1.55	1.19	−1.002	0.763	N/A
Advanced malignant tumor	N/A	N/A	−0.613	−2.631	−0.631	1.419
Neuroendocrine tumor	0.686	N/A	N/A	−1.482	−0.867	2.2
Homologous recombination of cells	−1.949	−3.259	N/A	N/A	N/A	N/A
Meiosis I of oocytes	N/A	1.698	1.698	1.698	N/A	N/A
Meiosis I of female germ cells	N/A	1.698	1.698	1.698	N/A	N/A
Lymphohematopoietic neoplasia	N/A	2.119	N/A	0.855	2.019	N/A
Lymphohematopoietic cancer	N/A	2.072	N/A	1.076	1.825	N/A
S phase of connective tissue cells	N/A	N/A	N/A	−2.759	−2.19	N/A
Development of body trunk	−0.832	N/A	N/A	−3.706	−0.211	0.199
Non−hematological solid tumor	1.189	1.001	1.256	−1.169	−0.293	N/A
Ploidy of cells	0.592	1.594	0.986	1.409	0.32	N/A
Ploidy	0.162	1.425	0.893	1.685	0.702	N/A
Transcription	N/A	N/A	N/A	−2.715	−1.582	0.531
Neoplasia of cells	−0.824	1.117	−0.367	−1.493	−0.26	0.76
Nucleation of cells	1.741	0.042	0.684	0.616	1.702	N/A
Abdominal neoplasm	−0.115	−0.417	0.078	−1.245	−0.642	2.19
Carcinoma	0.805	0.706	0.744	−1.178	0.328	0.919

Cytokinesis of cervical cancer cell lines	-2.607	-1.067	N/A	-0.998	N/A	N/A
Development of benign tumor	N/A	N/A	0.306	-0.94	0.965	2.415
Aneuploidy of cells	N/A	1.624	1.082	1.875	N/A	N/A
Skin cancer	N/A	N/A	N/A	-1.474	-1.128	1.958
Atrophy of genital organ	1.882	N/A	N/A	N/A	2.662	N/A
Adenoma	N/A	1.148	-0.441	-0.517	0.222	2.2
Cancer	N/A	1.864	1.499	-0.445	0.633	N/A
Breast or ovarian cancer	1.206	0.714	0.728	0.669	-1.066	N/A
Thoracic cancer	1.145	1.037	1.791	-0.166	0.231	N/A
S phase of tumor cell lines	N/A	-2.246	-2.112	N/A	N/A	N/A
Contractility of skeletal muscle	2.236	N/A	N/A	N/A	N/A	-2.121
Sensitivity of cells	0.719	N/A	N/A	1.536	2.102	N/A
G2/M phase	-0.9	-1.982	-0.956	-0.5	N/A	N/A
Breast cancer	1.188	0.692	0.714	0.637	-1.1	N/A
Cancer of cells	-0.571	1.132	-0.084	-1.21	0.585	0.748
Gastrointestinal tumor	0.555	1.029	0.647	0	0.078	1.96
Liver lesion	-0.88	-0.969	N/A	-0.352	0.556	1.494
Hypoplasia of organ	N/A	N/A	N/A	N/A	4.244	N/A
Nonhematologic malignant neoplasm	1.256	0.804	1.369	-0.771	-0.003	N/A
Transcription of RNA	N/A	N/A	N/A	-2.414	-1.231	0.553
S phase of bone cancer cell lines	-1.982	N/A	-2.2	N/A	N/A	N/A
Interphase of bone cancer cell lines	-1.982	N/A	-2.2	N/A	N/A	N/A
Lymphatic system tumor	N/A	1.344	0.979	0.259	1.458	N/A
Entry into interphase of oocytes	N/A	-2	-2	N/A	N/A	N/A
Death of embryo	N/A	N/A	3.988	N/A	N/A	N/A
Mitosis of tumor cell lines	N/A	-1.093	-0.861	-1.169	-0.817	N/A
Fibrogenesis	1.114	N/A	N/A	-1.115	-0.67	-1.014
Homologous recombination of DNA	-2.155	N/A	N/A	N/A	-1.734	N/A
Expression of RNA	N/A	N/A	N/A	-2.188	-1.694	N/A
Meiosis of germ cells	1.091	0.053	0.388	0.794	-1.446	N/A
Breast or gynecological cancer	1.206	0.714	0.728	0.399	-0.711	N/A
Malignant genitourinary solid tumor	1.206	0.633	1.348	0.057	-0.16	-0.283
Solid tumor	N/A	0.728	0.955	-0.887	-1.061	N/A
Lymphoreticular neoplasm	N/A	N/A	N/A	1.431	2.19	N/A

Renal lesion	N/A	0.762	N/A	1.265	1.311		-0.283
Gastrointestinal tract cancer	N/A	-0.896	-0.447	-1.066	-1.195	N/A	
Benign Tumors	N/A	1.139	-0.274	-1.331	0.854	N/A	
Abdominal adenocarcinoma	N/A	N/A	1.982	0.762	0.849	N/A	
Formation of filaments	1.36	N/A	N/A	-0.622	N/A		-1.572
G1 phase	N/A	-0.892	-0.828	-1.242	-0.567	N/A	
Meiosis I	N/A	0.882	0.882	0.882	-0.818	N/A	
Meiosis I of germ cells	N/A	0.882	0.882	0.882	-0.818	N/A	
Formation of muscle	N/A	N/A	N/A	-2.2	N/A		1.253
Skin lesion	-0.194	N/A	N/A	0.609	0.64		1.993
Acute leukemia	N/A	N/A	N/A	1.81	1.608	N/A	
Cancer of secretory structure	N/A	1.067	0.762	-0.478	-1.091	N/A	
Amplification of centrosome	N/A	N/A	-1.331	-2.026	N/A	N/A	
Colorectal tumor	N/A	-1.067	N/A	-0.896	-1.387	N/A	
Large intestine neoplasm	N/A	-1.067	N/A	-0.896	-1.387	N/A	
Myeloid leukemia	N/A	N/A	N/A	1.765	1.533	N/A	
G2 phase	0.374	-1.253	-0.58	0.308	0.775	N/A	
Acute myeloid leukemia	N/A	N/A	N/A	1.747	1.505	N/A	
Interphase of embryonic cell lines	N/A	N/A	-1.432	-0.99	-0.808	N/A	
Breast or colorectal cancer	0.751	-0.146	0.29	0.244	-1.719	N/A	
Atrophy of gonad	N/A	N/A	N/A	N/A	3.063	N/A	
Polyploidization of cells	N/A	0.69	0.69	0.971	0.702	N/A	
Sarcoma	N/A	N/A	1.437	0.758	0.831	N/A	
Genitourinary tumor	0.937	0.215	0.454	0.013	-1.398	N/A	
Colorectal cancer	N/A	-1.067	N/A	-0.555	-1.387	N/A	
Malignant neoplasm of large intestine	N/A	-1.067	N/A	-0.555	-1.387	N/A	
Anogenital cancer	1.206	0.323	1.026	-0.169	-0.282	N/A	
Cell viability of connective tissue cells	N/A	N/A	N/A	-2	-0.984	N/A	
Meiosis of oocytes	0.577	0.522	0.928	0.928	N/A	N/A	
Meiosis of female germ cells	0.577	0.522	0.928	0.928	N/A	N/A	
Interphase of connective tissue cells	N/A	N/A	N/A	-2.923	N/A	N/A	
Differentiation of epithelial tissue	N/A	N/A	N/A	1.516	-0.233		-1.107
Endocrine gland tumor	N/A	0.621	N/A	-1.362	-0.788	N/A	
Nonpituitary endocrine tumor	N/A	1.344	-0.415	-0.365	-0.641	N/A	

Interphase of fibroblasts	N/A	N/A	N/A	-2.746	N/A	N/A
Interphase of tumor cell lines	N/A	N/A	-1.62	-0.993	-0.122	N/A
Transcription of DNA	N/A	N/A	N/A	-1.52	-1.197	N/A
Quantity of centrosome	N/A	0.614	-0.29	0.566	1.238	N/A
Breast or ovarian carcinoma	1.154	0.651	0.339	0.355	-0.2	N/A
Breast carcinoma	1.154	0.651	0.339	0.355	-0.2	N/A
Lymphoma	N/A	N/A	N/A	0.968	1.726	N/A
Advanced stage tumor	N/A	N/A	-0.613	N/A	-0.631	1.419
Cell viability of fibroblasts	N/A	N/A	N/A	-2.647	N/A	N/A
Connective or soft tissue tumor	N/A	N/A	1.408	0.744	0.494	N/A
Skin tumor	N/A	N/A	N/A	-0.399	-0.046	2.17
Development of digestive organ tumor	N/A	0.326	1.044	-0.376	0.858	N/A
Fibrosis	N/A	N/A	N/A	N/A	-0.059	2.502
Spindle checkpoint of cervical cancer cell lines	N/A	-0.555	N/A	N/A	-1.982	N/A
Spindle checkpoint of tumor cell lines	N/A	-0.555	N/A	N/A	-1.982	N/A
Benign lesion	N/A	1.043	-0.136	-0.489	0.855	N/A
Metastatic solid tumor	N/A	N/A	0.413	-1.187	-0.917	N/A
Connective tissue tumor	N/A	N/A	1.111	0.543	0.833	N/A
Cell death of tumor cells	N/A	N/A	N/A	N/A	2.472	N/A
Advanced malignant solid tumor	N/A	-0.633	0.695	N/A	-1.119	N/A
Breakage of chromosomes	2.433	N/A	N/A	N/A	N/A	N/A
Quantity of chromosomes	0	0	-0.447	0.788	-1.134	N/A
Advanced extracranial solid tumor	N/A	N/A	1.154	N/A	-1.178	N/A
Cell death of cancer cells	N/A	N/A	N/A	N/A	2.286	N/A
Cell viability of colorectal cancer cell lines	N/A	N/A	N/A	N/A	-2.244	N/A
Hyperplasia of genital organ	2.236	N/A	N/A	N/A	N/A	N/A
Quantity of rib	N/A	N/A	N/A	N/A	N/A	2.213
Heart rate	N/A	N/A	N/A	N/A	N/A	-2.207
Mitosis of fibroblast cell lines	N/A	N/A	-2.207	N/A	N/A	N/A
Urogenital cancer	N/A	0.633	1.348	0.057	-0.16	N/A
Damage of muscle	N/A	N/A	N/A	N/A	N/A	2.194
Formation of brain	N/A	N/A	N/A	-2.191	N/A	N/A
Interphase of gonadal cells	N/A	N/A	-2.183	N/A	N/A	N/A
Mitosis of cervical cancer cell lines	N/A	-0.388	-0.44	-0.877	-0.478	N/A

Cell death of sarcoma cell lines	N/A	N/A	1.349	0.802	-0.012	N/A
Squamous-cell carcinoma	N/A	N/A	N/A	-1.067	-1.067	N/A
Quantity of glycogen	N/A	N/A	N/A	N/A	N/A	2.123
Proliferation of smooth muscle cells	N/A	N/A	N/A	N/A	-2.101	N/A
Ploidy of embryonic cell lines	N/A	N/A	N/A	1.383	-0.7	N/A
Necrosis of muscle	N/A	N/A	N/A	N/A	N/A	2.074
Cell viability of myeloma cell lines	N/A	N/A	N/A	N/A	-2.072	N/A
Cell viability of prostate cancer cell lines	N/A	N/A	N/A	N/A	-2.043	N/A
G2 phase of tumor cell lines	0.689	N/A	0.064	1.276	N/A	N/A
Transport of ion	N/A	N/A	N/A	N/A	N/A	-2.016
Transport of molecule	N/A	N/A	N/A	N/A	N/A	-1.998
Proliferation of vascular smooth muscle cells	N/A	N/A	N/A	N/A	-1.986	N/A
Nonsquamous non-small cell lung carcinoma	N/A	N/A	N/A	1.984	N/A	N/A
Contraction of striated muscle	N/A	1.982	N/A	N/A	N/A	N/A
Contraction of heart	N/A	N/A	N/A	N/A	N/A	-1.964
Differentiation of epithelial cells	N/A	N/A	N/A	1.694	0.263	N/A
Transformation of lumbar vertebra	N/A	N/A	N/A	N/A	N/A	1.948
Development of genital tumor	N/A	N/A	N/A	-0.555	1.387	N/A
Senescence of fibroblast cell lines	N/A	N/A	N/A	1.923	N/A	N/A
Formation of muscle cells	N/A	N/A	N/A	N/A	N/A	1.919
Leukemia	N/A	N/A	N/A	1.907	N/A	N/A
Cell death of breast cancer cell lines	0.683	N/A	N/A	1.045	0.08	N/A
Malignant myeloid neoplasm	N/A	N/A	N/A	1.795	N/A	N/A
Separation of centrosome	N/A	-1.741	N/A	N/A	N/A	N/A
G1 phase of embryonic cell lines	N/A	N/A	N/A	-0.956	-0.762	N/A
Epithelial neoplasm	N/A	N/A	N/A	-1.4	0.301	N/A
Bone marrow neoplasm	N/A	N/A	N/A	1.681	N/A	N/A
Myeloid neoplasm	N/A	N/A	N/A	1.681	N/A	N/A
Organ Degeneration	N/A	N/A	N/A	N/A	N/A	1.674
Homologous recombination repair of tumor cell lines	N/A	N/A	N/A	-1.669	N/A	N/A
Liquid tumor	N/A	N/A	N/A	1.647	N/A	N/A
Double-stranded DNA break repair	N/A	-0.254	-0.674	-0.218	0.492	N/A
Polyploidization	N/A	0.427	0.427	0.734	N/A	N/A
Development of central nervous system	N/A	N/A	N/A	-1.56	N/A	N/A

Conformational modification of DNA	N/A	N/A	-1.477	N/A	N/A	N/A
Microcephaly	N/A	N/A	N/A	1.477	N/A	N/A
Flux of ion	N/A	N/A	N/A	N/A	N/A	-1.465
Endocrine carcinoma	N/A	N/A	N/A	-1.067	-0.391	N/A
Bone marrow cancer	N/A	N/A	N/A	1.45	N/A	N/A
Cell cycle progression of tumor cell lines	N/A	N/A	0.282	-0.907	0.249	N/A
Senescence of fibroblasts	1.432	N/A	N/A	N/A	N/A	N/A
Function of muscle	N/A	N/A	N/A	N/A	N/A	-1.423
Homologous recombination repair of sarcoma cell lines	N/A	N/A	N/A	-1.294	0.128	N/A
Homologous recombination repair of bone cancer cell lines	N/A	N/A	N/A	-1.294	0.128	N/A
DNA damage response of cells	N/A	0.295	0.617	0.295	0.2	N/A
Quantity of mitotic spindle	N/A	N/A	0.765	0.637	N/A	N/A
Malignant neoplasm of retroperitoneum	N/A	N/A	N/A	1	-0.391	N/A
Edema of lung	N/A	N/A	N/A	N/A	N/A	1.387
Quantity of leptin in blood	N/A	N/A	N/A	N/A	1.383	N/A
Motor dysfunction or movement disorder	N/A	N/A	N/A	N/A	N/A	1.342
Transformation of vertebrae	N/A	N/A	N/A	N/A	N/A	1.329
Formation of RAD51 nuclear focus	N/A	N/A	N/A	-1.254	N/A	N/A
Extraadrenal retroperitoneal tumor	N/A	N/A	N/A	0.905	0.154	-0.152
Mitotic catastrophe of cervical cancer cell lines	N/A	N/A	N/A	N/A	-1.199	N/A
Apoptosis of male germ cells	1.183	N/A	N/A	N/A	N/A	N/A
Prostatic tumor	N/A	N/A	N/A	-1.032	0.147	N/A
Advanced lung cancer	N/A	N/A	N/A	N/A	-1.178	N/A
Renal tumor	N/A	N/A	N/A	1	0.152	N/A
Dysfunction of tumor cell lines	N/A	N/A	N/A	0.978	0.158	N/A
Quantity of microtubules	N/A	N/A	1.134	N/A	N/A	N/A
Function of cardiac muscle	N/A	N/A	N/A	N/A	N/A	-1.116
Brain lesion	N/A	N/A	N/A	N/A	N/A	1.067
Mitotic index	N/A	N/A	-1.048	N/A	N/A	N/A
Multinucleation of cells	N/A	0.042	0.66	0.341	N/A	N/A
Development of sarcoma	N/A	N/A	N/A	1.024	N/A	N/A
Edema	N/A	N/A	N/A	N/A	N/A	1.011
Dysfunction of heart	1	N/A	N/A	N/A	N/A	N/A
Macrocytic anemia	1	N/A	N/A	N/A	N/A	N/A

Extrapancreatic neuroendocrine tumor	N/A	N/A	N/A	−0.837	−0.16	N/A
Dysfunction of cells	0.6	N/A	N/A	N/A	0.394	N/A
Blue round small cell tumor	N/A	N/A	N/A	0.266	0.692	N/A
Metabolism of nucleic acid component or derivative	N/A	N/A	N/A	N/A	N/A	0.914
Activation of DNA endogenous promoter	N/A	N/A	N/A	−0.914	N/A	N/A
Cell death of myeloma cell lines	N/A	N/A	N/A	N/A	N/A	0.905
Failure of heart	N/A	N/A	N/A	N/A	N/A	0.896
Cancer of head	N/A	N/A	N/A	0.891	N/A	N/A
Multinucleation of cervical cancer cell lines	N/A	−0.342	0.436	0.104	N/A	N/A
Quantity of metal	N/A	N/A	N/A	N/A	N/A	0.879
Influx of Ca2+	N/A	N/A	N/A	N/A	N/A	−0.876
Head and neck cancer	N/A	0.664	N/A	0.21	0	N/A
Mitotic catastrophe of tumor cell lines	N/A	N/A	N/A	0.731	−0.135	N/A
Neoplasia of epithelial cells	N/A	0.391	N/A	−0.342	−0.128	N/A
Obesity	N/A	N/A	N/A	N/A	N/A	−0.847
Progression of tumor	N/A	N/A	N/A	N/A	−0.847	N/A
Mitotic catastrophe	N/A	N/A	−0.063	0.745	−0.031	N/A
Ploidy of epithelial cells	N/A	0	N/A	−0.378	0.447	N/A
Cell death of bone cancer cell lines	N/A	N/A	N/A	0.508	0.291	N/A
G1 phase of fibroblast cell lines	N/A	N/A	N/A	N/A	−0.762	N/A
Respiratory failure	N/A	N/A	N/A	N/A	N/A	0.762
Malignant connective or soft tissue neoplasm	N/A	N/A	N/A	0.758	N/A	N/A
Ion homeostasis of cells	N/A	N/A	N/A	N/A	N/A	−0.741
Urinary tract tumor	N/A	N/A	N/A	0.447	−0.277	N/A
Melanoma	N/A	N/A	N/A	−0.387	−0.332	N/A
Cell proliferation of breast cell lines	N/A	N/A	N/A	N/A	N/A	−0.713
Development of body axis	N/A	N/A	N/A	N/A	N/A	−0.686
Acute lymphoblastic leukemia	N/A	N/A	N/A	0.655	N/A	N/A
Ploidy of colorectal cancer cell lines	N/A	N/A	N/A	N/A	0.655	N/A
G1 phase of tumor cell lines	N/A	N/A	N/A	N/A	0.647	N/A
Nucleation of tumor cell lines	N/A	N/A	0.6	N/A	N/A	N/A
Apoptosis of breast cancer cell lines	N/A	N/A	N/A	N/A	−0.571	N/A
Metabolism of polysaccharide	N/A	N/A	N/A	N/A	N/A	0.57
Relaxation of muscle	0.555	N/A	N/A	N/A	N/A	N/A

Genital tract cancer	N/A	N/A	N/A	-0.555	N/A	N/A
Accumulation of tumor cell lines	N/A	N/A	N/A	N/A	0.555	N/A
Congenital malformation of genitourinary system	N/A	N/A	N/A	N/A	N/A	0.555
Pelvic cancer	N/A	N/A	N/A	-0.555	N/A	N/A
Polyploidy of cells	N/A	N/A	N/A	N/A	0.536	N/A
Quantity of metal ion	N/A	N/A	N/A	N/A	N/A	0.503
Binding of chromosome components	N/A	N/A	-0.496	N/A	N/A	N/A
Pancreatobiliary tumor	N/A	N/A	N/A	-0.113	-0.377	N/A
Quantity of Ca2+	N/A	N/A	N/A	N/A	N/A	0.462
Male genital neoplasm	N/A	N/A	N/A	-0.32	0.129	N/A
Atrophy of muscle	-0.447	N/A	N/A	N/A	N/A	N/A
Hereditary connective tissue disorder	N/A	N/A	N/A	0.447	N/A	N/A
Thyroid gland tumor	N/A	N/A	N/A	N/A	-0.415	N/A
Neck neoplasm	N/A	N/A	N/A	N/A	-0.415	N/A
Anemia	0.41	N/A	N/A	N/A	N/A	N/A
Differentiation of muscle	N/A	N/A	N/A	N/A	N/A	0.393
Pancreatic tumor	N/A	N/A	N/A	0.378	N/A	N/A
Apoptosis of bone cancer cell lines	N/A	N/A	N/A	0.307	-0.04	N/A
Lung injury	N/A	N/A	N/A	N/A	N/A	0.314
Litter size	N/A	N/A	N/A	N/A	N/A	-0.308
Concentration of lipid	N/A	N/A	N/A	N/A	0.296	N/A
Nervous system neoplasm	0.277	N/A	N/A	N/A	N/A	N/A
B-cell neoplasm	N/A	N/A	N/A	-0.263	N/A	N/A
Differentiation of muscle cells	N/A	N/A	N/A	N/A	N/A	0.26
Breast adenocarcinoma	N/A	N/A	N/A	N/A	0.254	N/A
Differentiation of embryonic tissue	N/A	N/A	N/A	N/A	N/A	0.24
Catabolism of polysaccharide	N/A	N/A	N/A	N/A	N/A	0.218
Quantity of nucleus	N/A	N/A	N/A	-0.194	N/A	N/A
Concentration of triacylglycerol	N/A	N/A	N/A	N/A	0.165	N/A
Renal cancer	N/A	N/A	N/A	N/A	0.152	N/A
Damage of axons	N/A	N/A	N/A	N/A	0.152	N/A
Poikilocytosis	0.152	N/A	N/A	N/A	N/A	N/A
Erythrocytosis	0.152	N/A	N/A	N/A	N/A	N/A
Formation of mammary gland	N/A	N/A	N/A	N/A	N/A	-0.152

Urinary tract cancer	N/A	N/A	N/A	N/A	0.152	N/A
Colon tumor	N/A	N/A	N/A	−0.152	N/A	N/A
Breakage of DNA	0.128	N/A	N/A	N/A	N/A	N/A
Cellular homeostasis	N/A	N/A	N/A	N/A	N/A	−0.109
Differentiation of myoblasts	N/A	N/A	N/A	N/A	N/A	0.086
Apoptosis of sarcoma cell lines	N/A	N/A	N/A	N/A	0.023	N/A
