# Supplementary material for: Novel predator-induced phenotypic plasticity by hemoglobin and physiological changes in the brain of Xenopus tropicalis
Source: Front Physiol. 2023 Jun 6;14:1178869. doi: 10.3389/fphys.2023.1178869 (PMC10279953; doi:10.3389/fphys.2023.1178869)
Supplement: Supplementary file 6 [file Table3.pdf]

Supplementary Table 3

Factor loadings for PCA, and stands for number of Ex group as 1:contrl, 2:10 days, 3:5-days out, 4: 48 hours, 5: 24 hours, respectively.

Ex group	First Component	Second Component	Third Component	Fourth Component	Fifth Component
1	-0.94478	0.66506	-0.58724	1.64373	-0.21176
1	-0.45479	1.23088	0.26187	2.48672	0.30339
1	1.70109	-0.1424	1.50561	0.77763	-0.41585
1	-0.0869	1.5807	-0.26335	2.13192	-0.56476
1	0.1797	1.1234	-0.43728	1.08273	-0.35712
1	0.90239	-1.04715	0.33356	-1.08568	2.10539
1	2.14063	-0.72336	0.37925	-0.00972	1.19374
1	0.55273	-0.53075	-0.36898	-0.07353	-0.05873
1	1.24384	2.25772	-0.24466	0.22827	3.20623
1	2.60277	0.1131	0.46053	0.87317	1.25374
1	-0.43134	0.41437	-0.32581	-0.72594	2.63087
1	-0.389	-0.25174	-0.45317	0.05824	1.08248
1	-0.03659	0.93205	-0.80858	-0.30994	0.57615
1	0.65438	1.44773	-0.15752	-0.18447	0.94461
1	0.57074	0.4181	-0.03345	-0.1493	1.42201
1	0.99292	-0.06653	0.51584	0.32062	0.12763
1	0.61076	0.22685	1.0077	1.1456	1.07183
1	-0.62667	1.31855	-0.73041	-0.87977	-0.72323
1	-0.2686	-0.051	-0.55609	-1.18216	0.75223
1	-0.23969	0.98139	-0.70088	-0.07629	0.23415
1	-0.34798	-0.48419	-0.02374	-0.47436	0.59505
1	2.09313	1.14857	0.35907	-0.27532	-0.73985
1	0.47651	1.6287	-0.27322	-0.90634	0.42657
1	-0.29029	0.01657	0.26145	0.1955	-0.72737
1	0.82608	1.03753	-0.85062	-0.62585	0.18776
1	-0.22701	0.86035	-0.58219	0.31266	0.40542
1	1.77239	-0.6868	0.15964	-0.0505	0.54754
1	0.49509	-0.10922	-0.38951	0.01473	-1.20465
1	-0.61791	-0.7813	0.10465	2.38807	-0.18416
1	-0.06265	0.31104	-0.44122	0.40368	1.54137
2	1.69193	-0.70749	0.17777	0.8233	-0.45322
2	-0.49009	1.43783	-0.15401	-0.20361	-1.33858
2	-0.38647	-0.80681	0.06695	0.47875	-1.38783
2	-0.74382	0.34713	-0.58281	-0.30655	-0.35267
2	-0.37702	0.01	0.39733	0.98836	-0.84978
2	0.76161	0.06784	-0.02549	-0.9277	-0.51801
2	2.43248	-0.66086	0.06684	-1.01771	-1.01477
2	-0.26693	-0.08022	-0.32533	0.61391	0.62501
2	0.55371	-1.51364	0.32357	-1.49189	0.31489
2	0.23934	-0.43758	1.02307	-0.95854	0.43149
2	0.46224	-0.23672	1.80136	-0.18213	-0.62788
2	-1.14443	-0.22537	-0.73618	-1.80743	-0.85367
2	0.35	-0.78294	-1.09478	-1.50896	-1.71119
2	-1.95206	-1.27617	-1.02772	-0.49108	0.11658
2	-0.31605	1.15793	-0.5408	-1.26591	-1.98856
2	0.03141	-0.27651	-0.51589	-0.63969	-1.8976
2	-0.98776	-0.50511	-1.49215	-0.00545	1.20405
2	-1.33904	0.77708	-0.76722	0.60738	-0.72512

2	−1.47932	−0.80674	−0.91366	−0.51907	0.63348
2	−0.27744	0.44425	−1.90671	−0.98357	−1.26485
2	0.22248	−1.26887	−0.31357	−0.60255	0.14564
2	0.10647	−0.64853	−0.37114	−2.22449	−0.83637
2	0.64284	−1.86786	−0.29969	−0.54938	0.60305
2	−1.36798	−1.34867	−0.36595	0.29597	0.4215
2	0.70628	−0.83237	−0.65345	0.50246	−1.41787
2	−1.77509	0.09016	−1.09686	−0.12283	−0.51921
2	−1.16713	1.71902	−0.54198	0.34095	−1.78962
2	−1.39412	0.31446	−1.15317	−0.88178	0.2174
2	−0.66226	1.3876	−0.65114	−0.34836	−0.71048
2	0.60391	0.60785	0.00769	0.93874	0.42815
2	0.11407	−0.05265	1.41559	0.92223	0.48912
2	1.68111	1.95667	−1.03693	−0.28324	−0.97419
2	−0.71421	−0.60401	0.14721	1.03298	−0.13622
2	0.58554	−0.44577	−0.77512	−1.37343	−0.64279
2	0.61236	−0.53191	−0.08559	−0.64503	−0.7227
2	−0.37512	0.62937	−0.25983	−1.62692	0.31852
2	0.16828	0.34647	−0.47068	−2.1047	0.21602
2	−1.06921	0.99888	−0.65184	0.15164	1.06693
2	−1.19885	−0.2962	−1.11476	−1.03154	−1.62235
2	−1.15503	1.95527	−0.14346	1.041	0.25056
2	−0.26175	2.57912	−0.58255	−0.0331	−1.20912
2	1.24583	−0.91303	0.3545	−0.28261	−1.55041
2	−0.11864	−0.45848	0.1046	−0.56592	0.74019
2	1.45126	0.02805	−0.85892	−2.06833	−0.7503
2	0.28154	0.70718	0.66744	−1.42696	0.57782
2	0.08706	−0.60546	−0.53341	0.30726	0.4784
2	−0.27986	0.46319	−0.51725	−1.16526	−0.18727
2	−0.5311	−0.53226	0.00186	−0.26122	0.07742
2	−0.33445	−0.84535	0.85047	−0.6685	0.2337
2	0.34112	0.43016	−0.11274	0.05571	−0.52768
2	0.6243	1.36326	0.30906	−1.0218	−0.28251
2	−0.54122	0.33961	0.11285	−0.79216	0.37749
2	0.12146	−0.35691	0.82001	−1.76954	1.25166
2	0.77122	0.87142	−2.05243	−0.13082	0.21545
2	−1.21117	0.09955	−1.65392	−2.5624	0.65852
2	−0.62495	0.4219	−1.12469	−1.70689	0.76807
2	−0.9142	−0.69681	0.39467	−1.02056	−0.89798
2	−0.77167	−2.49163	−0.72813	1.70975	−0.391
2	0.31025	0.24894	0.16055	1.46411	0.15277
2	0.81532	0.14548	−1.08546	−0.99165	0.04779
3	0.73125	−0.14573	1.07784	0.49377	−0.94834
3	1.33127	−0.90046	0.86906	0.44171	−0.80912
3	1.01812	1.60373	0.3219	0.48091	0.39887
3	0.59522	−0.11046	−0.11206	1.53796	−0.82168
3	0.45776	1.05754	−0.44648	0.82875	−0.86367
3	1.31094	0.42667	0.28055	0.16224	−0.54004
3	0.29882	−0.55427	0.27433	−0.01027	0.19766
3	1.41291	1.07574	−0.31691	−0.96167	−0.40206
3	0.49009	−0.12135	−0.49646	−0.25639	0.8867
3	0.84376	2.14712	−0.45442	0.22747	1.35643

3	1.26958	−0.18645	−0.52372	−1.85552	0.64555
3	0.6949	0.50553	0.85023	0.4332	1.27868
3	0.12077	0.49948	−0.75616	0.27288	−2.15154
3	0.58977	−0.11575	−0.47626	−1.0899	−0.49069
3	2.3622	−0.13437	−0.72654	−2.30601	−0.80209
3	0.33343	−1.93229	0.13538	1.60095	−0.26333
3	1.76744	−0.48584	0.86588	−1.88026	0.14877
3	0.09827	−0.30238	0.79133	−1.2365	0.11934
3	0.26464	−0.24914	−0.44297	−1.78808	1.10413
3	0.30986	−1.55604	−0.5034	0.09283	0.48539
3	−1.16725	1.43647	−0.34338	0.18279	0.41772
3	−0.85307	1.28974	−0.84213	−0.41038	2.16517
3	0.02875	1.13717	−1.71331	−0.12339	−0.73536
3	−1.20322	0.95914	−0.61354	−0.09064	0.20366
3	−0.22765	0.63813	−0.82712	−0.32938	0.79017
3	0.22763	−0.50174	−1.40622	−0.98896	−0.68029
3	−0.91875	−1.27868	1.26245	−1.19276	0.25574
4	0.60165	1.10008	0.92256	0.91375	−0.65078
4	0.55088	−0.28986	1.34992	−0.11002	−1.20191
4	1.07225	0.12201	−0.45602	−0.02766	−1.18331
4	1.18234	−0.06364	0.55932	0.23667	−0.35528
4	0.34982	0.7139	0.13791	0.67871	−0.06801
4	0.99086	0.2853	−0.15162	2.42124	0.5546
4	0.84289	−0.5301	1.04016	0.79438	−0.97639
4	0.54321	0.71499	0.14166	1.48599	−1.11243
4	0.47487	−0.16656	0.91112	1.56906	−2.1317
4	−0.41989	2.1774	−1.24727	2.02566	−1.26444
4	0.60078	0.3198	−0.88821	1.13072	−1.42974
4	1.78873	−0.13038	−0.59539	0.78806	0.11183
4	1.18277	−0.73639	1.10825	0.23947	0.30899
4	2.5374	−1.733	0.62347	0.49962	0.14035
4	0.37548	0.76072	−0.52791	0.70656	−0.97796
4	−1.4352	1.1359	2.91625	−1.29848	−0.95361
4	−0.4436	−0.67316	3.62648	−0.38804	−0.27109
4	−0.21335	1.53081	3.99916	−0.53699	1.04956
4	−0.07027	1.62813	2.84244	−0.4157	0.05471
4	−1.23663	−0.3063	2.25493	−1.29885	0.31914
4	−1.49586	−0.68957	3.18229	−0.4652	−0.80835
4	−0.84031	1.20755	1.92051	−0.57955	1.00946
4	−1.93807	0.0055	2.81838	−0.55676	0.64264
4	−1.9445	1.79402	3.10842	−0.90488	−0.72169
4	−2.17166	−0.24036	0.99695	−1.2309	−0.15736
4	−1.25099	0.82883	1.48144	−0.22885	0.91401
4	−1.44642	0.505	2.11626	0.44843	0.80316
4	−1.44367	1.05708	2.48857	−0.39793	−0.71791
5	1.23826	1.20012	0.51457	0.55749	−1.66311
5	−0.23962	−0.00647	0.7956	0.64101	0.83534
5	0.67292	−0.32553	1.25337	−0.32902	−0.46132
5	1.60163	−0.7436	0.96105	0.84231	0.59057
5	0.51459	−0.45851	0.29418	0.02333	−0.8881
5	1.76066	−0.63	0.00643	0.38202	0.47753
5	0.36797	−1.01621	−0.66459	0.66353	−0.54993

5	0.49349	0.17818	0.11775	0.7298	0.05544
5	0.07979	0.4921	−0.56957	0.49368	0.48268
5	−0.9084	−1.68096	−1.00865	1.66108	0.06879
5	−0.09662	0.03044	−0.38715	1.29695	−0.95763
5	0.51228	−0.1539	0.18645	0.06494	−0.62947
5	−0.15317	−2.37447	0.48653	0.96567	0.20988
5	−1.52655	−0.68722	−0.38813	0.59185	0.28614
5	−1.28237	−0.94824	−0.64437	0.31298	1.24319
5	0.3882	−1.00076	−0.23633	0.89113	−0.16353
5	0.34129	−1.0819	−0.34117	0.46138	1.14218
5	0.10439	−0.23058	−0.12365	1.28951	4.17791
5	−1.01131	−1.11884	0.35215	0.64304	1.29202
5	−1.14237	−0.60215	0.47584	2.24589	0.03208
5	0.35855	0.83704	−0.83823	0.1151	1.31563
5	−0.84164	−0.6122	−0.08367	0.89815	0.68385
5	0.39379	−2.7009	0.2303	0.29388	0.69168
5	−0.41971	−1.46603	0.20953	−0.56055	−0.54184
5	0.71869	−1.04972	−0.6752	0.60861	1.49789
5	0.42601	−2.35691	−1.0465	0.23208	0.74554
5	−1.13767	−1.12386	−1.09356	−0.52354	−0.88894
5	−1.59866	−1.08441	−0.89996	−0.79262	−0.48157
5	−1.67304	−1.43558	−0.67441	0.09069	−1.21905
5	−0.69991	−0.00122	−0.26069	1.60483	1.31565
5	−1.93482	−0.42813	−0.2184	0.98416	−2.41557
5	−1.49836	−0.6215	−0.92787	1.2376	−0.37432
5	0.03884	0.08249	−0.30444	0.6716	−1.41929
5	−0.70549	0.15655	0.2515	2.0517	−0.29762
5	0.95478	−0.84952	0.37284	−0.74762	0.31674
5	−0.82485	−1.96196	−0.004	1.03484	−0.23655
5	−0.11153	1.54619	−1.0312	0.7508	2.30265
5	−0.09565	1.21357	−0.04667	1.11451	−0.49144
5	−1.44869	−2.41779	0.6507	−0.29021	−0.8652
5	−1.31243	−0.3506	−1.19725	−0.83272	1.45843
5	−1.59586	−0.21934	−0.39876	0.69065	0.22192
5	−1.08173	0.40911	−1.26241	0.45998	1.67791
5	−0.26623	−0.55286	−0.68899	0.73212	1.299
5	−0.86838	−0.0541	−0.55815	−0.74373	−0.07896
