# Supplementary material for: Novel predator-induced phenotypic plasticity by hemoglobin and physiological changes in the brain of Xenopus tropicalis
Source: Front Physiol. 2023 Jun 6;14:1178869. doi: 10.3389/fphys.2023.1178869 (PMC10279953; doi:10.3389/fphys.2023.1178869)
Supplement: Supplementary file 7 [file DataSheet1.pdf]

Supplementary Methods

1.1 Experimental animals and design

The *Xenopus tropicalis* tadpoles used in this experiment were procured from an inbred line prepared by the Amphibian Research Center, Hiroshima University. Approximately 1,500–1,800 tadpoles were reared in the breeding room. Prior to the experiment, approximately 200–300 tadpoles of similar size (stage 51) were selected and placed in two 20 L tanks (45 × 15 × 30 cm). Weak tadpoles were removed at this stage. Subsequently, 20 tadpoles of similar size (stage 51) were randomly chosen from the holding tank based on the *Xenopus laevis* stage series (https://www.xenbase.org/anatomy/alldev.do) and placed at a concentration of 20 per aquarium in 2 L glass aquaria (25 × 10 × 8 cm) for acclimation. The water used in this experiment was treated with activated charcoal CW130B (Tsurumi Coal, Kanagawa, Japan), and 2 mL of Tetra Perfect Water (Tetra, Melle, Germany) was also added to the 10 L of water in the aquaria. Tadpoles were fed Sera Micron (Sera, Heinsberg, Germany), reared at 25 °C during the experimental period, and selected for experimentation via a uniform randomization protocol described by Mori et al. (2017). Wild larval salamanders (*Hynobius retardatus*) were obtained from Nayoro and Asahikawa ponds in Hokkaido, Japan (Supplementary Figure 1), placed in separate 12 L aquaria, and fed with mosquito larvae (*Tubifex* spp.) ad libitum until they reached a length of 4 cm. All experiments were conducted in the laboratory at 25 °C, under a natural day/night regime (14/10 h). The complete experimental protocol is summarized in Fig. 1a. For experimental treatment, six predator exposure conditions were used, including a non-exposed control: control, predator exposure for 6 h; (Ex 6hr), 24 h; (Ex 24hr), 48 h; (Ex 48hr), 10 days; (Ex 10day), and Ex 5day-Out treatment, where the tadpoles were exposed to predators for five days and then reared in the absence of predators for another five days. Eighteen aquaria were set up for predation-exposed and control tadpoles, while an additional three, one, one, one, and two aquaria were set up as backups for the Ex 10day, Ex 48hr, Ex 24hr, Ex 6hr, and 5day-Out groups, respectively. Accordingly, each treatment group had three replicates, and a variable number of backups were maintained under predation pressure. All aquaria (including backups) were randomly arranged and separated by cardboard blinders.

The predation experiments were initiated by introducing one salamander larva into an aquarium containing 20 tadpoles. To minimize the loss of tadpoles during predation experiments, we replaced the salamander larvae daily with others kept in a holding tank with ad libitum access to *Tubifex* larvae (Mori et al., 2017). Replacement predators were chosen at random from each holding tank, and the number of tadpoles in each aquarium was counted daily. After 10 days, the tadpoles were collected from each experimental group and placed in small transparent plastic cases. Overhead and lateral view photographs of the tadpoles were used to measure the 15 body parameters (Fig. 1b, Supplementary Table 1) via ImageJ (ver1.53e) (Schneider et al., 2012). The tadpoles were weighed after being dried with a paper towel. The mean values of the first and second components for principal component analysis (PCA) were determined using all data. Mean measurements among the control, Ex 24hr, Ex 48hr, Ex 10day, and Ex 5day-Out groups are shown in Supplementary Figure 2.

1.2 Statistical analysis of PCA results

MANOVA statistical analysis was performed on the results of the PCA, followed by multiple comparisons of the five components. The homogeneity of variance for each of the five components was tested via Leven statistics. Components for which the test of homogeneity of variance was rejected

were tested with Dunnet T3, and those for which homogeneity of variance was maintained were tested via Bonferroni post hoc tests. Detailed results are provided in Supplementary Table 4.

1.3 Library preparation and sequencing

RNA was extracted from the *X. tropicalis* tadpole brains using Monarch (New England BiolLabs Japan) and sent to Beijing Genomics Institute (BGI) group for library preparation and sequencing. Library preparation and sequencing of extracted total RNA were performed by the BGI Group (Shenzhen, China) in accordance with their in-house protocol. Following DNase I treatment, mRNA was isolated using magnetic beads with oligo dT and fragmented by mixing with a fragmentation buffer. These mRNA fragments were used as templates for cDNA preparation. Short fragments were purified using elution buffer for end reparation as well as single nucleotide A (adenine) addition and were connected with adapters. Thereafter, suitable fragments (approximately 200 bp) were selected as templates for PCR amplification. The prepared sample library was quantitatively and qualitatively analyzed using the 2100 Bioanalyzer (Agilent Technologies, CA, USA) and StepOnePlus Real-Time PCR System (Thermo Fisher Scientific, MA, USA). Finally, the library was sequenced using HiSeq X Ten (Illumina, CA, USA).

1.4 Transcriptome analysis using RNA-seq and ingenuity pathway analysis (IPA)

Read data were filtered, trimmed, cleaned, quality-checked using AfterQC v.2.7 (Chen et al., 2017), and then mapped to the reference genome of *X. tropicalis* (Xenopus_tropicalis.JGI_4.2.dna.toplevel.fa) derived from Ensemble using HISAT2 v.2.1.0 (Pertea et al., 2016). Sequence alignment/map (SAM) files produced after mapping were sorted and converted into binary alignment/map (BAM) files using SAMtools v.1.9 (Takeuchi et al., 2016). The number of reads mapped to each gene was counted using StringTie v.1.3.4 (Pertea et al., 2016) against an annotation file (Xenopus_tropicalis.JGI_4.2.92.gtf) derived from Ensemble. Differential expression analysis was performed using the R-based package EdgeR v.3.8.6 (https://bioconductor.org/packages/release/bioc/html/edgeR.html) (Robinson et al., 2010), which filters out low-expression genes, while genes with > 1 count per million (CPM) in at least two samples were retained. After normalizing the trimmed mean of the M-values, the differentially expressed genes (DEGs) were extracted from pairwise comparisons of the control vs. Ex 24hr and Ex 48hr experimental groups using an exact test with a false discovery rate (FDR) <0.05 and visualized through a heat map (Fig. 2a). The pathway signaling analysis software IPA (Qiagen; Hilden, Germany) was used to identify the functional networks of genes expressed in the brain. To perform IPA analysis, we converted the Ensembl IDs of *X. tropicalis* genes to those corresponding to *Homo sapiens*. CPM fold changes of Ex 6hr, Ex 24hr, Ex 48hr, and Ex 10days compared to the control as well as of the Ex 5day-Out compared to the Ex 10days group were calculated using equations (S1) and (S2), respectively:

log2(CPM Ex 6 hr/Ex 24 hr/ Ex 48 hr / Ex 10 days) − log2(CPM control + 1) (S1)

log2(Ex5day-Out +1) − log2(CPM control or Ex 10 days +1) (S2)

Genes with > 1.5-fold expression changes were analyzed using IPA (Content *v*.48207413), while IDs were consolidated using the expression value set as "average" and measurements for resolving duplicates set as "Ex Log Ratio." A total of 1364 IDs were obtained, of which 1362 were mapped based on sequence homology with *H. sapiens*. Signal transduction pathways were identified using IPA of 1341 genes (after eliminating the duplicates).

1.5 Starch and sucrose metabolism via PYGM, G6PC1, PDK4, and G6PC/G6PC2/G6PC3

IPA analysis was performed to investigate the expression of genes related to starch and sucrose metabolism based on RNA-seq data in Fig. 2b

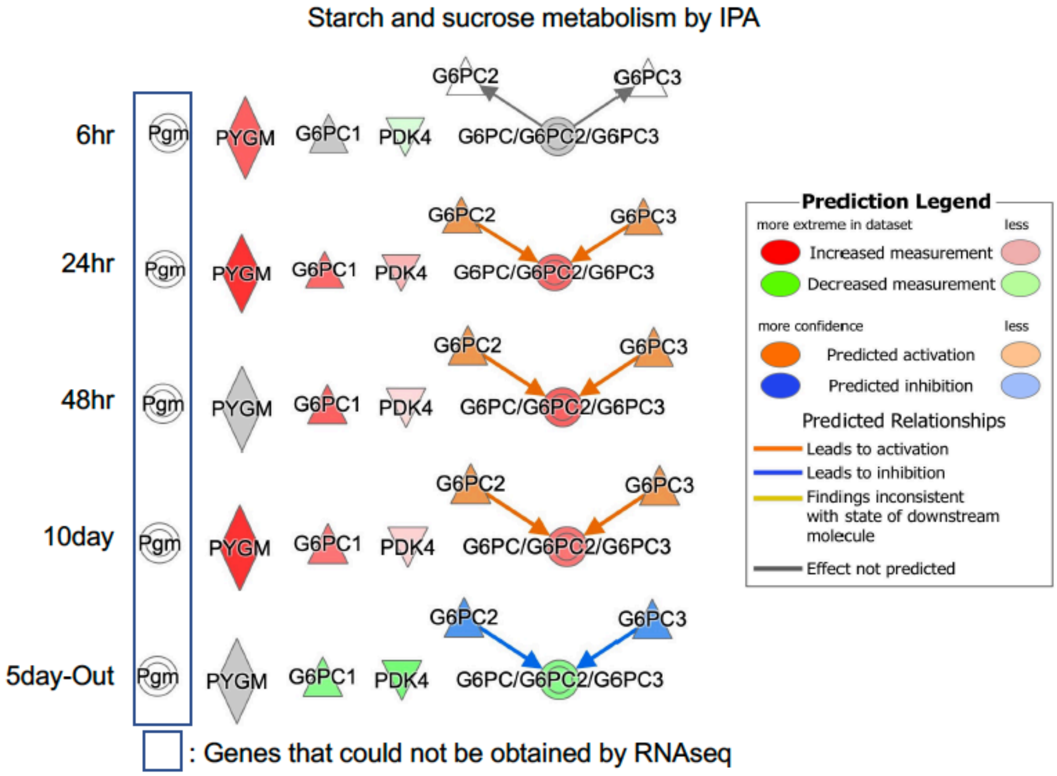

1.6 Visualization of hemoglobin using a hyperspectral camera

Images were acquired at 3.55× magnification using a Leica MZ16 microscope (Tokyo, Japan) with two halogen lamps (Leica CLS 150 XD) as light sources (Fig.3a). The visualization of hemoglobin was completed by analyzing hyperspectral images using the normalized difference vegetation index (Lausch et al., 2013), as per equation (S3):

$$\text{NDVI} = \frac{(IR-R)}{(IR+R)} \cdots\cdots\cdots\cdots \quad \text{(S3)}$$

where R is the reflectance of hemoglobin, and IR is the reflectance of near-infrared color. As the arterial region of *Xenopus* larvae showed specific spectral absorption at 570–575 nm, R was taken as 570–575 nm and IR as 750 nm. The heat map was created by averaging the spectral reflectance with R at 570–575 nm and IR at 750–800 nm.

Oxygen-hemoglobin in the tadpole brain was analyzed using methods described by a former report (Tetschke et al., 2016) to calculate oxygen saturation using normalized absorption spectra. The oxygen-

sensitive wavelength at 600 nm [AbsNormx;y (600 nm)] and the oxygen-insensitive wavelength at 570 nm [AbsNormx;y (570 nm)] were measured. The absorbance ratio (AR) is inversely proportional to the oxygen saturation level within the brain (denoted as sO_2), where sO specifies HbO_2 (Equation (S4)):

$$ARx,y = \frac{AbsNormx,y(600nm)}{AbsNormx,y(570nm)} \fallingdotseq \frac{1}{sO2} \cdots\cdots\cdots\cdots \quad \text{(S4)}$$

As the ratio of oxygen-bound to free hemoglobin can be calculated using this equation, the ratio of sO_2 was calculated from the calibration curve of AR and sO_2 (%), in accordance with a previous report (Tetschke et al., 2016), and a heat map was generated (Fig.3c). The ratio of oxygen bound to free hemoglobin was calculated from the heat map by identifying areas in the brain where sO_2 was more strongly produced.

1.7 Capillary electrophoresis time-of-flight *mass* spectrometry (CE-TOF-MS) analysis of brains from *X. tropicalis* tadpoles

Tadpoles were anesthetized in an ice water bath and dissected immediately. The brains were excised under a microscope, placed in a tube, immediately frozen using liquid nitrogen, and stored at −80 °C until experiments. Two brains were combined into a single sample, and three samples were treated as a single experimental group. A 50 % solution of acetonitrile (v/v; standard internal concentration, 10 µM; Human Metabolome Technologies, Inc., Tsuruoka, Japan) was added to the samples, which were then crushed (at 1500 rpm for 120 s, twice) using a crusher, and centrifuged at 2300 × *g* at 4 °C for 5 min. After centrifugation, 400 µL of the upper layer was transferred to an ultrafiltration tube (Ultrafree MC PLHCC, HMT, 5 kDa centrifugal filter unit). The filtrate was dried and dissolved in 50 µL Milli-Q water for capillary electrophoresis mass spectrometry analysis as per the procedure specified by Soga et al. (2006). The authors declare that the research contained within this manuscript complied with the Animal Welfare Guidelines for Journal Publication. All animal housing and experimental protocols were in compliance with the Japanese Government Animal Protection and Management Law (No. 105) as well as the Japanese Government Notification on Feeding and Safekeeping of Animals (No. 6).

1.8 Real-time polymerase chain reaction (PCR)

RNA was extracted from twelve brains for each treatment group using the RNA Extract Mini Kit (Qiagen) according to manufacturer's instructions, and the quality of the total RNA was assessed as described for RNA-seq. One microgram of total RNA was reverse-transcribed into cDNA using a QuantiTect Reverse Transcription Kit (Qiagen) in accordance with the manufacturer's protocol. Subsequently, the prepared cDNA was quantified and diluted to a concentration of 300 ng mL^{-1} for use as a template in the real-time PCR. The reaction mixture contained the following components: SYBR Green master mix (10 µL), forward and reverse primers (0.64 µL; 10 µM each), template cDNA (300 ng), and RNase-free water; the final volume of the reaction was 20 µL. Appropriate housekeeping genes from the RNA-seq data were used as internal controls, and genes with a correlation coefficient (R) > 0.98 were screened based on the cycle threshold (CT) values. The validity of the primer design was evaluated by ensuring an amplification efficiency of 0.85–1.15 %. We identified stromal cell factor 4 (*scf4*), as the CT values for this transcript were identical among the treatment groups with five different dilutions (4-, 8-, 16-, 32-, and 64-fold). Real-time PCR was performed on a Rotor-Gene thermal cycler (Qiagen) to determine the expression profiles of superoxide dismutase 3 (*sod3*), phosphoserine phosphatase (*psph*), glucose-6-phosphatase catalytic subunit 3 (*g6pc3*), hemoglobin

subunit alpha-3 (*hba3*), 5′-aminolevulinate synthase 2 (*alas2*), fumarylacetoacetate hydrolase (*fah*), phosphoserine aminotransferase 1 (*psat*), and glycogen phosphorylase muscle-associated (*pygm*). The primers used to amplify these genes are summarized in Supplementary Table 2. Real-time PCR was performed for 35 cycles under the following conditions: incubation at 95 °C for 5 min, annealing at 95 °C for 5 s, and extension at 60 °C for 10 s. Transcript levels were calculated as per the ΔΔCT method (Livak and Schmittgen 2001).

References

Chen, S., Huang, T., Zhou, Y., Han, Y., Xu, M., and Gu, J. (2017.) AfterQC: Automatic filtering, trimming, error removing and quality control for fastq data. BMC Bioinform. 18, 80. doi:10.1186/s12859-017-1469-3.

Lausch, A., Pause, M., Doktor, D., Preidl, S., and Schulz, K. (2013). Monitoring and assessing of landscape heterogeneity at different scales. Environ. Monit. Assess. 185, 9419-9434.

Livak, K.J., and Schmittgen, T.D. (2001). Analysis of relative gene expression data using real-time quantitative PCR and the 2(-Delta Delta C(T)) Method. Methods. 25, 402-408.

Mori, T., Yanagisawa, Y., Kitani, Y., Yamamoto, G., Goto-Inoue, N., Kimura, T., et al. (2017). The constant threat from a non-native predator increases tail muscle and fast-start swimming performance in Xenopus tadpoles. Biol. Open. 6, 1726-1733.

Pertea, M., Kim, D., Pertea, G.M., Leek, J.T., and Salzberg, S.L. (2016). Transcript-level expression analysis of RNA-seq experiments with HISAT, StringTie and Ballgown. Nat. Protoc. 11, 1650-1667.

Robinson, M.D., McCarthy, D.J., and Smyth, G.K. (2010). edgeR: A bioconductor package for differential expression analysis of digital gene expression data. Bioinformatics. 26, 139-140.

Schneider, C.A., Rasband, W.S., and Eliceiri, K.W. (2012). NIH Image to ImageJ: 25 years of image analysis. Nat. Methods. 9, 671-675.

Soga, T., Baran, R., Suematsu, M., Ueno, Y., Ikeda, S., Sakurakawa, T., et al. (2006). Differential metabolomics reveals ophthalmic acid as an oxidative stress biomarker indicating hepatic glutathione consumption. J. Biol. Chem. 281, 16768-16776.

Takeuchi, T., Yamada, A., Aoki, T., and Nishimura, K. (2016) cljam: a library for handling DNA sequence alignment/map (SAM) with parallel processing. Source Code Biol. Med. 11, 12.

Tetschke, F., Markgraf, W., Gransow, M., Koch, S., Thiele, C., Kulcke, A., et al. (2016). Hyperspectral imaging for monitoring oxygen saturation levels during normothermic kidney perfusion. J. Sens. Sens. Syst. 5, 313-318.

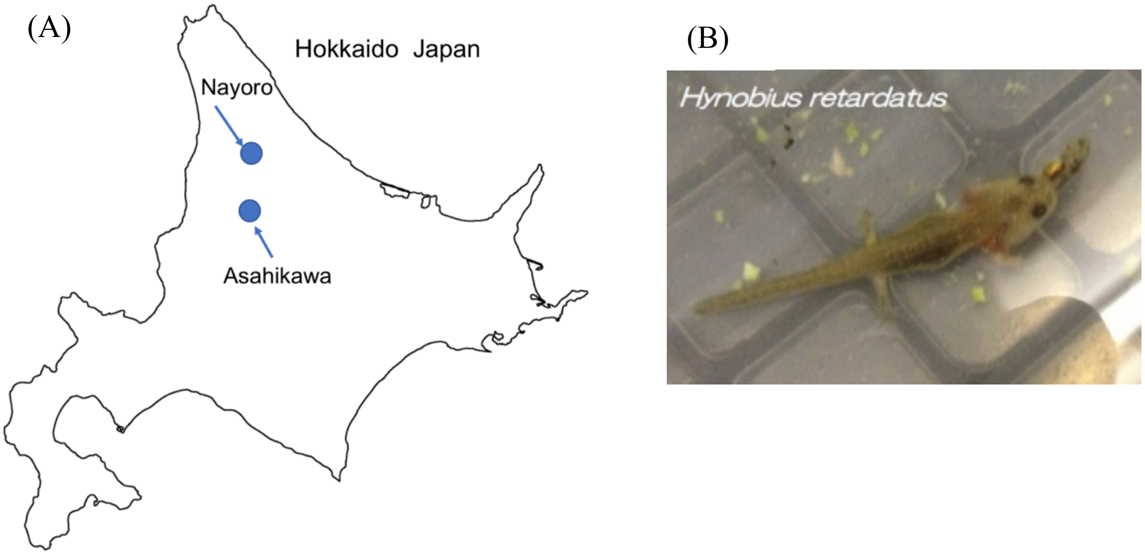

Supplementary Figure 1. Map showing the sampling sites (A) for larval salamanders (*Hynobius retardatus*) (B).

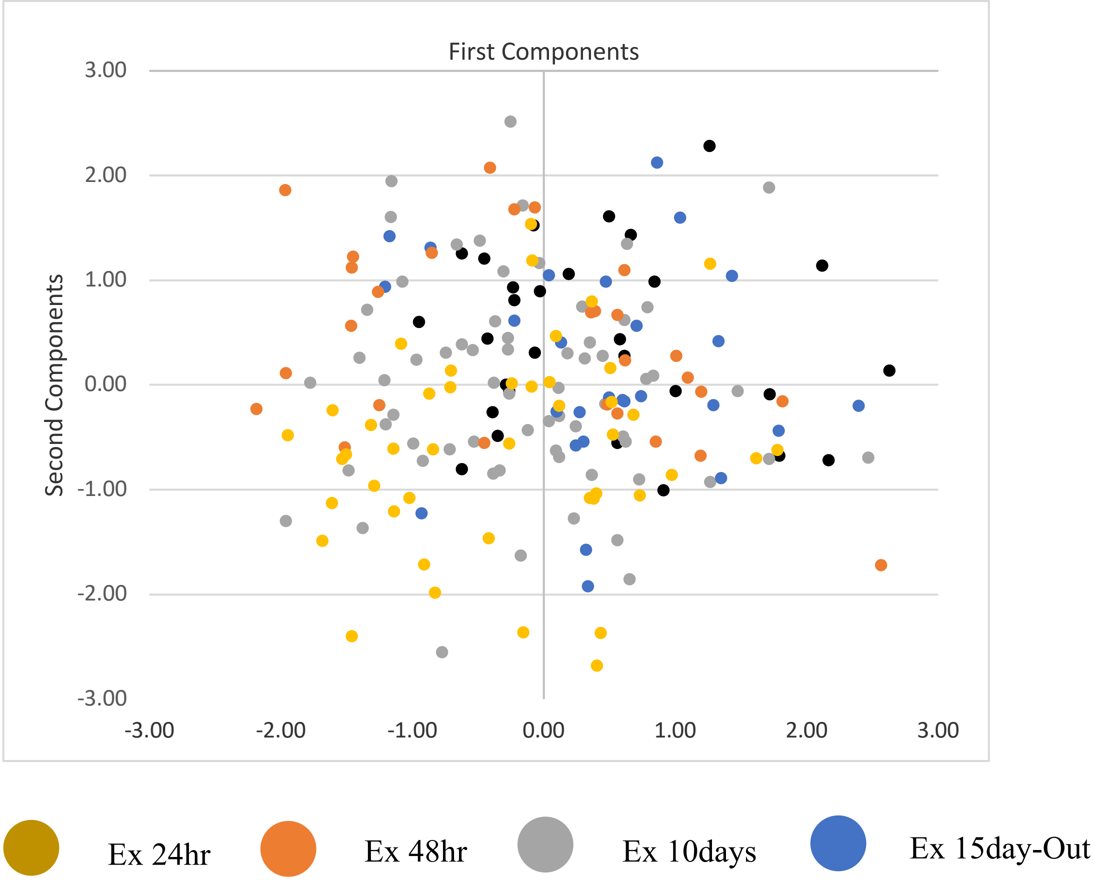

Supplementary Figure 2. Principal component analysis (PCA). Fifteen body parts of *X. tropicalis* were measured in the five experimental groups, including controls. Relationships between the measured variables and the five groups were subjected to PCA using SPSS (version 20).
