# Supplementary material for: Novel predator-induced phenotypic plasticity by hemoglobin and physiological changes in the brain of Xenopus tropicalis
Source: Front Physiol. 2023 Jun 6;14:1178869. doi: 10.3389/fphys.2023.1178869 (PMC10279953; doi:10.3389/fphys.2023.1178869)
Supplement: Supplementary file 8 [file Table7.pdf]

Supplementary Table7. Top tox functions in the brain from X. tropicalis predicted by IPA under various predation conditions.

Name	P-value Range	Molecules	Gene Name
6 hr			
Increased Levels of Hematocrit	2.80E-06 - 2.80E-06	9	HBZ,ERFE,PER2,EPB42,MYB,BDKRB2,APOE,SCARB1,SLC4A1
Increased Levels of Red Blood Cells	1.86E-03 - 1.86E-03	6	HBZ,EPB42,CDK2,SCARB1,EPOR,SLC4A1
Increased Levels of Creatinine	1.56E-01 - 1.29E-01	2	GATM,SLC4A1
Increased Levels of LDH	1.35E-0.1 - 1.35E-01	1	FOXM1
Decreased Levels of Albumin	1.45E-0.1 - 1.45E-01	1	BDKRB2
24 hr			
Increased Levels of Red Blood Cells	1.67E-03 - 1.67E-03	9	HBZ,EZH2,CTF1,SLC7A1,CDK2,SCARB1,PRKAG1,HGF,EPOR
Increased Levels of Hematocrit	3.94E-02 - 3.94E-02	6	HBZ,ERFE,LIG1,APOE,SCARB1,PRKAG1
Increased Levels of LDH	2.67E-01 - 2.67E-01	1	FOXM1
Increased Levels of Creatinine	3.84E-01 - 3.03E-01	1	EDNRB
Increased Levels of AST	3.39E-01 - 3.39E-01	1	APOE
48 hr			
Increased Levels of Red Blood Cells	1.61E-03 - 1.61E-03	7	HBZ,EZH2,CTF1,CDK2,SCARB1,HGF,EPOR
Increased Levels of Hematocrit	7.10E-02 - 7.10E-02	4	HBZ,MYB,LIG1,SCARB1
Increased Levels of LDH	2.38E-01 - 1.06E-01	2	FOXM1,ITGB2
Increased Levels of Alkaline Phosphatase	3.12E-01 - 3.12E-01	2	BMP6,CCN2
Increased Levels of ALT	3.19E-01 - 3.19E-01	1	ITGB2
10 days			
Increased Levels of Red Blood Cells	1.94E-02 - 1.94E-02	11	HBZ,EZH2,CTF1,SLC7A1,FANCC,HOXB4,CDK2,MDM2,SCARB1,PRKAG1,EPOR
Increased Levels of Creatinine	7.10E-02 - 7.10E-02	6	GAMT,ARHGDIA,EDNRB,CCN2,GATM,MDM2
Increased Levels of Hematocrit	2.38E-01 - 1.06E-01	7	HBZ,PER2,MYB,LIG1,SCARB1,PRKAG1,NR2C2
Decreased Levels of Albumin	3.12E-01 - 3.12E-01	1	AKT1
Increased Levels of Alkaline Phosphatase	3.19E-01 - 3.19E-01	5	LDLR,DLL1,AKT1,NOTCH1,CCN2
5 day-Out			
Increased Levels of Red Blood Cells	3..24E-04 - 3.24E-04	11	EZH2,CTF1,FANCC,HOXB4,CDK2,MDM2,SCARB1,PRKAG1,HGF,EPOR,SLC4A1
Increased Levels of Hematocrit	2.45E-03 - 2.45E-03	9	ERFE,PER2,LIG1,BDKRB2,APOE,SCARB1,PRKAG1,NR2C2,SLC4A1
Increased Levels of ALT	4.99E-03 - 4.99E-03	4	NCF1,ITGB2,APOE,C1QA
Increased Levels of Creatinine	1.98E-01 - 6.26E-02	3	MDM2,KL,SLC4A1
Decreased Levels of Albumin	3.23E-01 - 8.35E-02	3	AKT1,BDKRB2,KL
